# Developmental deficits of MGE-derived interneurons in the *Cntnap2* knockout mouse model of autism spectrum disorder

**DOI:** 10.3389/fcell.2023.1112062

**Published:** 2023-02-01

**Authors:** Noorya Yasmin Ahmed, Rhys Knowles, Lixinyu Liu, Yiming Yan, Xiaohan Li, Ulrike Schumann, Yumeng Wang, Yovina Sontani, Nathan Reynolds, Riccardo Natoli, Jiayu Wen, Isabel Del Pino, Da Mi, Nathalie Dehorter

**Affiliations:** ^1^ The Australian National University, The John Curtin School of Medical Research, Canberra, ACT, Australia; ^2^ Tsinghua-Peking Center for Life Sciences, IDG/McGovern Institute for Brain Research, School of Life Sciences, Tsinghua University, Beijing, China; ^3^ Institute of Neurosciences, Spanish National Research Council (CSIC), Sant Joan d’Alacant, Spain

**Keywords:** interneuron, maturation, striatum, autism, CNTNAP2

## Abstract

Interneurons are fundamental cells for maintaining the excitation-inhibition balance in the brain in health and disease. While interneurons have been shown to play a key role in the pathophysiology of autism spectrum disorder (ASD) in adult mice, little is known about how their maturation is altered in the developing striatum in ASD. Here, we aimed to track striatal developing interneurons and elucidate the molecular and physiological alterations in the *Cntnap2* knockout mouse model. Using Stereo-seq and single-cell RNA sequencing data, we first characterized the pattern of expression of *Cntnap2* in the adult brain and at embryonic stages in the medial ganglionic eminence (MGE), a transitory structure producing most cortical and striatal interneurons. We found that *Cntnap2* is enriched in the striatum, compared to the cortex, particularly in the developing striatal cholinergic interneurons. We then revealed enhanced MGE-derived cell proliferation, followed by increased cell loss during the canonical window of developmental cell death in the *Cntnap2* knockout mice. We uncovered specific cellular and molecular alterations in the developing Lhx6-expressing cholinergic interneurons of the striatum, which impacts interneuron firing properties during the first postnatal week. Overall, our work unveils some of the mechanisms underlying the shift in the developmental trajectory of striatal interneurons which greatly contribute to the ASD pathogenesis.

## Introduction

Interneurons are vital cells that regulate the balance between excitation and inhibition in the brain (Dehorter et al., 2017; Antoine et al., 2019) and are required to shape functional circuits during development (Fuccillo, 2016). Deficits to interneurons have been identified in both human patients and in mouse models of neurodevelopmental disorders such as Autism Spectrum Disorder (ASD). Interneuron dysfunction supports behavioural traits including impaired social interactions and repetitive behaviours (Fuccillo, 2016; Contractor et al., 2021), Therefore, these cells present interesting targets for therapeutic interventions (Selimbeyoglu et al., 2017). Yet, how the maturation of interneurons is affected at embryonic and early postnatal stages in this condition has remained poorly investigated and as such, the cellular mechanisms contributing to ASD pathophysiology is still unknown.

Most of the interneurons fated to the cortex and striatum derive from a transitory embryonic brain structure, the Medial Ganglionic Eminence (MGE; (Penagarikano et al., 2011; Vogt et al., 2017; Knowles et al., 2021), where they express specific molecules, such as the Lim Homeodomain Lhx6 and NK2 homeobox 1 (Nkx2.1) transcription factors (Knowles et al., 2021). Between embryonic day (E) 12.5 and postnatal day (P) 10, a combination of innate genetic programs (Yap and Greenberg, 2018), external environmental cues, and neuronal activity shape interneuron specification and facilitate the establishment of functional cells (Babij and De Marco Garcia, 2016; Mayer et al., 2018; Wong et al., 2018). The ASD risk gene Contactin-associated protein-like 2 (Cntnap2/CASPR2), first shown to be altered in patients presenting with severe autism features (Strauss et al., 2006), is a transmembrane scaffolding protein and a cell adhesion protein of the neurexin family which coordinates several processes that are critical for the maturation of neuronal circuits (Benamer et al., 2020), including myelination (Scott et al., 2017), cell excitability (Poliak et al., 1999) and synapse development (Varea et al., 2015). Whilst Cntnap2 cell-autonomously affects adult interneuron functioning (Vogt et al., 2017; Antoine et al., 2019) and has been shown to be expressed from E14 in the MGE (Penagarikano et al., 2011; Vogt et al., 2017), the specific pattern of expression in the brain, as well as its influence on interneuron development have remained poorly investigated; in particular, how the absence of *Cntnap2* perturbs the maturation of the MGE-derived interneurons.

Although they account for ∼5% of the total neuronal population, striatal interneurons are functionally diverse and consist of two main cell types, i.e. the Lhx6-expressing GABAergic interneurons and the Lhx6-expressing cholinergic interneurons (Lozovaya et al., 2018; Ahmed et al., 2021). These two populations precisely regulate the striatal output neurons and present with characteristic electrophysiological profiles. The Lhx6-expressing GABAergic interneurons are fast-spiking cells, characterized by short duration action potentials with large and rapid kinetics (Tepper et al., 2010), whilst the cholinergic cells are tonic active neurons that display adaptation and prominent after-hyperpolarizations (Ahmed et al., 2019).

Here, we hypothesized that the developmental trajectory of these striatal interneurons is perturbed in the *Cntnap2* Knockout (KO) mouse model of ASD (Penagarikano et al., 2011). We analyzed the cellular and molecular developmental alterations of the developing MGE-derived interneurons of the striatum at embryonic and early postnatal stages, in control and *Cntnap2* KO mice. We show that interneuron production, migration and integration in the developing striatum are affected in the *Cntnap2* KO mouse model, likely contributing to ASD-related pathogenesis.

## Material and methods

### Mice

*Lhx6-iCre;td-Tomato* (“Control”) and *Cntnap2* ^*−/−*^ *; Lhx6-iCre; td-Tomato* (“*Cntnap2* KO”) male and female mice (*Cntnap2* ^−/−^, Jackson ID: #017482, (Tepper et al., 2010)) were used. To track MGE-derived cells through the stages of embryonic development, we also utilized the inducible *Nkx2.1CreER:TdTomato* and *Nkx2.1CreER:TdTomato:Cntnap2* ^*−/−*^ mice. Tamoxifen in corn oil (40 mg/ml) was used to orally gavage pregnant female mice at E12.5 (3.3 μL/g, 30 mg/kg) to induce cre expression.

Timed matings were used to generate embryonic tissue for collection. Detection of a plug was considered embryonic day (E) 0.5. Embryonic tissue was collected at E12.5, E14.5, and E15.5 (a minimum of three embryos), and postnatal tissue was collected at postnatal day (P) 0, 4, 6, 10, and 30. All procedures were conducted in accordance with the Australian National University Animal Experimentation Ethics Committee (protocol numbers A2019/46 & A2021/43).

### Spatial transcriptomics (Stereo-seq)

To unbiasedly determine the expression pattern of *Cntnpa2* in the adult mouse brain, we employed Spatial Enhanced Resolution Omics-Sequencing (Stereo-seq) technology from BGI, in beta testing (V1.0, not commercially available). This analysis combines whole transcriptome information with nanoscale resolution. Upon interaction with tissue section, cDNA is synthesized *in situ* from mRNA captured by the chip probes. The spatial transcriptomic profile of the section was performed by cDNA sequencing with cell spatial coordinates.

An adult (P30+) brain was extracted and embedded and frozen in optimal cutting temperature compound (OCT), then sectioned sagittally *via* cryostat at a thickness of 10 µm. Tissue was mounted onto STOmics-GeneExpression-S1 chips (1 cm^2^), which are patterned grids of probes containing spatial coordinates. A permeabilization analysis was first performed to determine the optimal permeabilization time: tissue on the chip was fixed in methanol, then incubated at 37°C for various lengths of time (0–30 min) in Permeabilization Reagent (BGI, 0.01N HCl and PR Enzyme). Reverse transcription was then performed at 42°C for 1 + hours. The whole chip was then imaged (AxioScan Slide Scanner), and the permeabilization time with the strongest fluorescence signal with the lowest signal diffusion was selected. The gene expression experiment was then performed with duplicates. The tissue was fixed as before, permeabilized, and the reverse transcription performed. The tissue was removed from the chip, and cDNA Release Mix was added to the chip and incubated at 55°C for 3 + hours. The quality of the generated cDNA library was checked *via* Bioanalyser, after which we proceeded with one library (F2). Library preparation and sequencing was done at the WEHI core facility using the DNBSEQ-G400RS FCL PE100 kit and sequencer.

#### Mapping the raw data

Stereo-seq Analysis Workflow (SAW) software (Chen et al., 2021) was used to process the sequencing information. The coordinate ID (CID) sequences were mapped to the designed coordinates on the chip with a mismatch tolerance of 1 bp. The Molecular ID (MID) sequences with a quality score greater than 10 were kept. cDNA sequences were mapped to the reference genome (mm10) by STAR (Dobin et al., 2013). We obtained an expression profile matrix with 70901 units and 18191 features using a bin size 50×50 DNA nanoball (DNBs).

#### Unsupervised clustering

We used the *stereopy* packages (Chen et al., 2021) to perform the normalisation and unsupervised clustering. In the bin 50×50 DNBs expression matrix, the low-quality units with fewer than 100 genes and more than 10% mitochondria gene level were filtered out. The low-abundance genes that were expressed in fewer than 10 cells were removed. On the filtered cells, the expression matrix was normalised, and PCA was analyzed. Next, the Gaussian smoothing (Shen et al., 2022) method was performed to make the expression value closer to reality. On the smoothed values, we performed dimensionality reduction with the function “*stereo.tl.pca”*. The clusters were identified using the functions “*stereo.tl.neighbors”* and “*stereo.tl.leiden”*.

#### Clustering annotation

We extracted the top marker genes expressed in each cluster based on the most significant adjusted p-values using *“sc.findmarker”* function from *Scanpy* (Wolf et al., 2018). The clusters were annotated based on the known marker expression and spatial localisation of clusters in the brain. Tissue identities were assigned based on the above information.

#### Differential expression and pathway enrichment analysis

Differential expression (DE) analysis was conducted pairwise between clusters using the function “*FindMarker*” from Seurat (Stuart, 2019). The p-values and the adjusted p-values, and log2 fold-changes were calculated between two clusters. The differential expressed genes were selected with a adjusted p-values smaller than 0.01, log2 fold-change greater than 1. *ClusterProfiler* (Yu et al., 2012) was used to find enriched Gene Ontology (GO) terms from the DE genes.

#### Hotspot module analysis

Hotspot analysis (DeTomaso and Yosef, 2021) was carried out on the normalised data to identify the spatially functional modules with informative genes. Within each identified module, GO enrichment analysis was performed using *ClusterProfiler* (Yu et al., 2012).

### Analysis of published single-cell RNA sequencing data

Using the available single-cell RNA-sequencing data from Mi et al., 2018, we performed t-Distributed Stochastic Neighbor Embedding (t-SNE) analysis to visualize data in a two dimensional map, as in (22). We generated plots containing E12.5 and E14.5 MGE neurons, along with histograms showing the regional and temporal identities of cells. We made feature plots for *Cntnap2* expression in E12.5 and E14.5 MGE neurons and a heatmap plot in which we classified cell clusters into three classes (*i.e.* cortical interneurons, striatal interneurons and cholinergic neurons), according to the marker gene expression pattern. We then compared *Cntnap2* levels across three cell classes.

### Tissue collection

Pregnant females were cervically dislocated and the embryos were removed from the mother, and placed into ice-cold 0.01M phosphate buffered saline (PBS). E12-14 embryos were decapitated, and the brain was dissected and placed into 4% paraformaldehyde (PFA) for overnight fixation. Considering the larger size of the E15 embryos and to ensure adequate fixation we performed cardiac perfusion on E15.5 embryos. The arm of the embryo was removed to reveal the heart and the animal was perfused with 1 ml 0.01M PBS followed by 3 ml 4% PFA. The brain was then removed and fixed overnight in 4% PFA. Brains were then washed in 0.01M PBS for 5 h to remove the fixative. Embryonic tissue was embedded in 4% Bacto-Agar in 0.01M PBS heated to 37°C, allowed to cool, and then sectioned at 60 μm using a Leica 1000S vibratome and kept in a cryoprotective ethylene glycol solution at -20°C until further processing for immunohistochemistry.

Postnatal mice (P0-P30) were deeply anaesthetised on ice (P0-P4) or with 4–4.5% isoflurane (P6-P30) and perfused transcardially with 0.01M PBS followed by 4% PFA. Brains were post-fixed for 2–5 h in 4% PFA. The fixative was then washed from tissues 3 times for 30 min each with 0.01M PBS. Tissue was sectioned at 60 μm using a Leica 1000S vibratome and kept in a cryoprotective ethylene glycol solution at -20°C until further processing for immunohistochemistry.

### Immunohistochemistry

Sections were first washed twice for 5 min in 0.01M PBS and permeabilised twice for 5 min with 0.25% TritonX. Non-specific binding sites were then blocked by immersing the tissue in a blocking solution (10% Normal Donkey Serum, 0.25% TritonX, 2% Bovine Serum Albumin in 0.01M PBS), for 2 h at room temperature. Slices were then incubated in primary antibodies added to blocking solution overnight at 4°C on a shaker. The following day, slices were left at room temperature for 1 h on a shaker and were washed 3 times for 15 min each with 0.01M PBS. Slices were then stained with fluorescence conjugated or biotinylated secondary antibodies in blocking solution for 2–3 h and then washed 3 times for 15 min each with 0.01M PBS. This was repeated for those requiring tertiary antibodies for 1 h. Slices were then incubated with 5 μM DAPI (Sigma) for 10 min and washed 4 times for 5 min each with PBS. The slices were mounted on Livingstone slides submerged in gelatine, allowed to dry, covered with Mowiol (Sigma) and a coverslip (Thermofisher).

The following primary antibodies were used: mouse anti-Tuj1 (1:500, Biolegend), rabbit anti-Ki67 (1:500, Abcam), mouse anti-Lhx6 (1:300; Santa Cruz), goat anti-ChAT (1:200; Merck), rabbit anti-mgluR5 (1:50; Alomone), rabbit anti-cleaved Caspase-3 (1:200, Cell Signalling), rabbit anti-NMDAR2C (1:100, ThermoFisher); and secondary antibodies: donkey anti-rabbit 488 (1:200, Molecular Probes), anti-goat and anti-mouse Alexa 488, 555 or 647 (1:200; Life Technologies), and streptavidin 647 (1:200, Jackson).

To mark proliferating cells at specific developmental time points, pregnant female mice were injected intraperitoneally with 5-ethynyl 2′-deoxyuridine (EdU) dissolved in distilled water (5 mg/ml; 30 mg/kg). To label the EdU that was incorporated into the DNA of proliferating cells at the time of IP injection, the Click-It^®^ EdU Imaging Kit from Invitrogen was used, with slight modifications. To label nuclei, DAPI was used. In addition, washes were completed in 1x PBS instead of 3% BSA. To perform staining of EdU, tissue was washed in 1x PBS. The tissue was washed in 500 μL of 0.5% Triton for 15 min. The reaction cocktail was prepared, according to the Click-iT^®^ protocol. The triton solution was removed from the wells and replaced with the reaction cocktail solution for 30 min. The reaction cocktail was removed and replaced with blocking solution for 2 h at RT.

### Confocal imaging

Immunostained slices were imaged with a Nikon A1 confocal microscope controlled with the NIS-elements advanced imaging software or a Leica SP5 confocal microscope controlled by LAS AF. Images were collected at 4x, 10x, 20x, 40x, and 63x. To ensure consistency in imaging, the same confocal microscope was used within a data set and the parameters for offset, pinhole, gain and laser intensity were kept constant for each experimental set.

### Image analysis

Image analysis was largely completed using Fiji/ImageJ. To count Lhx6+ and Nkx2.1 + cells, a custom-made code was used to run multiple threshold events at different threshold values, whilst simultaneously completing image processing and detecting particles of the appropriate size and circularity. The code was modified for a number of parameters including the lower and upper threshold, number of thresholds, minimum and maximum cell size, and circularity, dependent on the dataset being analyzed. During analysis of control and experimental conditions, all parameters were kept consistent. VZ thickness was measured by creating a straight line on Fiji at the apex of the MGE spanning from the ventricular wall to the VZ/SVZ border, which was defined by Tuj1 immunostaining. The SVZ thickness was approximated by measuring from the VZ/SVZ border at the apex of the MGE through to the mantle, which was defined by either the higher level of Tuj1 and/or the lack of Ki67 cells.

To measure the thickness of the migratory streams, masks surrounding the superficial and deep migrating postmitotic cells were created on ImageJ manually. The width of the middle of the mask was measured in µm. The same masks were used for automated cell counting to quantify the total density of Nkx2.1 + cells.

The size of the postnatal striatum was quantified from low magnification images containing the whole striatum; the contour was traced and the area measured. The thickness of the postnatal PFC was measured as a line perpendicular to the midline, spanning across all cortical layers. Rostro-caudal coordinates were kept consistent between control and knockout conditions.

### Fluorescence activated cell sorting (FACS)

Mice were rapidly decapitated, the brain was removed and placed in an ice cold oxygenated sucrose-based cutting solution. 400 μm coronal slices were cut using a Leica VT1200S vibratome. The dorsal striatum was dissected from slices in an Artificial Cerebrospinal Fluid (ACSF) containing (in mM): 124 NaCl, 3 KCl, 2 CaCl2, 1 MgCl2, 1.25 NaH2PO4, 26 NaHCO3 and 10 glucose saturated with 95% O2 and 5% CO2. Tissue was disrupted using micro-knife and transferred to a 15 ml tube containing 10 ml digestion buffer (0.5g Trehalose and 10 mg Pronase, 1 mg DNAse I, 50 μL MgCl2 1M in oxygenated ACSF; Sigma) at 37°C for 30 min with inversion every 10 min. The tissue was allowed to settle on ice for 1 min, the digestion buffer was removed and the pellet was washed with 2 ml washing buffer (10 ml ACSF, 0.5 g Trehalose, 1 mg DNAse I, 50 μL MgCl2 1M in oxygenated ACSF) at 4°C. To dissociate the cells, the tissue was resuspended in 1 ml wash buffer and triturated 12–15 times with 1 ml pipette. Tubes were placed on ice, large tissue pieces were allowed to settle, and ∼500 μL of cloudy suspension containing dissociated cells was transferred to a 1.5 ml tube on ice. This process was repeated for the remaining 500 μL suspension with 200 μL pipette 12–15 times and pooled in to the 1.5 ml tubes on ice. The last cells were removed by adding 200 μL to the original tube. The cell suspension was filtered through a 70 μm thick mesh filter into a snap cap tube and 200 μL DAPI 5 μM was added to exclude dead cell. The cells were sorted on a BD FACSMelody Cell Sorter. The neuronal population was gated ensuring myelin and other debris were excluded by FSC/SSC profile. Doublets were then excluded by FSC-W/FSC-H and SSC-W/SSC-H. Dead cells shown as DAPI positive cells were also excluded. The Lhx6+ cell population was sorted by gating on TdTomato + signal above negative background from TdTomato negative mice. A fraction of the sorted cells were checked for purity by running some of the sorted cells back into the cell sorter, and were determined as 99% pure. The remaining cells were sorted into 350 μL RLT buffer (RNeasy Microkit; Qiagen) with 1% β-Mercaptoethanol. Sorted cells were immediately frozen on dry ice and placed at -80°C until further processing.

### Quantitative real time polymerase chain reaction (qRT-PCR)

Total RNA was extracted using the RNeasy Micro kit (Qiagen) for FACS sorted cells and some tissue samples and the RNeasy Mini Kit (Qiagen) for other tissue samples according to manufacturer’s instructions, including DNase treatment for some samples. RNA concentration and purity were determined using the Nanodrop spectrophotometer (Thermo Fisher Scientific). RNA was reverse transcribed into cDNA using SuperScriptIII First Strand Synthesis System (Invitrogen) with random hexanucleotides according to manufacturer instructions. cDNA was analyzed using real time qPCR, in technical triplicates using TaqManTM Gene Expression Assay. Reactions were performed on a MicroAmp™ Optical 96- or 384-Well Reaction Plate with Barcode (Applied Biosystems). Each 10 μL reaction (performed on 384-well plates) contained 5 μL of TaqManTM universal PCR Master Mix (Applied Biosystems), 0.5 μL of Taqman probes gene expression assay and 4.5 μL of cDNA (10 ng RNA). Each 20 μL reaction (performed on 96-well plates) contained 10 μL of TaqManTM universal PCR Master Mix (Applied Biosystems), 1 μL of Taqman probes gene expression assay and 4 μL of cDNA (10 ng RNA), and 5 μL of RNase free water. PCRs were monitored using the 7900HT Real-Time PCR system (Applied Biosystems) or the StepOnePlus™ Real-Time PCR system (Applied Biosystems). The reaction cycle involved incubation at 50°C for 2 min, at 95°C for 10 min to allow for AmpliTaq Gold DNA 57 Polymerase activation and then cycled 40 times at 95°C for 15 s (denaturation) and 60°C for 1 min (annealing and extension). Analysis was performed by comparing the experimental Ct values against those for the reference housekeeping gene Gapdh. A 1/ΔCt value was calculated to represent differences between conditions. The following Taqman primers were used: Lhx6 (Mm01333348_m1), ChAT (Mm01221880_m1), Grin2C (Mm00439180_m1), Grin2D (Mm00433822_m1), Grin 1 (Mm00433790_m1), Grin2A (Mm00433802_m1), Grin2B (Mm00433820_m1), Lhx8 (Mm00802919_m1), Gbx2 (Mm00494578_m1), Zic4 (Mm00657066_m1), Grm5 (Mm00690332_m1).

### Electrophysiology

Postnatal mice were deeply anaesthetised with 4–4.5% isoflurane and transcardially perfused with an ice-cold oxygenated, sucrose-based artificial cerebrospinal fluid (ACSF) containing (in mM): 248 sucrose, 3 KCl, 0.5 CaCl2, 4 MgCl2, 1.25 NaH2PO4, 26 NaHCO3, and 1 glucose, saturated with 95% O_2_ and 5% CO_2_. The animals were then decapitated, the brain was removed and placed in ice cold oxygenated sucrose-based ACSF cutting solution. 400 µm coronal slices were sectioned using a Leica VT1200S vibratome. Slices were then placed into room temperature ACSF containing (in mM): 124 NaCl, 3 KCl, 2 CaCl2, 1 MgCl2, 1.25 NaH2PO4, 26 NaHCO3 and 10 glucose saturated with 95% O2 and 5% CO2, where they were maintained until recording.

Slices were transferred to a chamber and continuously perfused with ACSF at 34°C. Red fluorescent protein (TdTomato) expressing Lhx6 cells located in the dorsal striatum were visualized by infrared-differential interference optics with a ×40 water-immersion objective. For targeting TdTomato expressing neurons, slices were illuminated by green light through the objective. Microelectrodes (4–6 MΩ) were pulled from borosilicate glass (1.5 mm outer diameter x 0.86 inner diameter) using a vertical P10 puller (Narishige).

For current-clamp recordings, we targeted *Lhx6-iCre;td-Tomato*-positive (Lhx6+) CINs, based on the large size of their soma and typical tonic firing properties (Ahmed et al., 2019), and putative Lhx6+ fast-spiking GABAergic cells and discarded the somatostatin cells, which present with elongated soma shape, very high input resistance (>600 MΩ), low threshold spike (LTS), and typical AHP kinetics and rebound firing (Tepper et al., 2010). We used a potassium-gluconate-based intracellular solution containing (in mM): 140 K-gluconate, 10 HEPES, 2 NaCl, 4 KCl, 4 ATP, and 0.4 GTP. Neurobiotin (2–5 mg/ml) was added for post-recording immunocytochemistry. For voltage-clamp recordings, a cesium gluconate-based intracellular solution was used containing (in mM): 120 Cs-gluconate, 13 CsCl, 1 CaCl2, 10 HEPES, and 10 EGTA (pH 7.2–7.4, 275–285 mOsm). We used the cesium-gluconate solution to measure spontaneous and miniature GABAA currents at the reversal potential for glutamatergic (+10 mV) events and glutamatergic currents at -60 mV.

Electrophysiological signals were low-pass filtered on-line at 10 kHz with a Multiclamp 700B (Axon Instruments) amplifier and acquired at a 20-kHz sampling rate with a LIH 8 + 8 (HEKA) data acquisition board and WinWCP software (created by John Dempster, University of Strathclyde). Circuit capacitance was corrected after gigaseal formation. Series resistance and liquid junction potential were not corrected. Following break-in, the test pulse was monitored for a few seconds to ensure a stable, low access resistance (Ra <20MΩ). To measure postsynaptic currents, the voltage was held at -70 mV for AMPA/KA or +10 mV for GABA_A_ events under NBQX and +40 mV under NBQX 10uM, GABAZINE 10uM and 4-AP (10uM; Sigma). PSC rise and decay times were calculated as the intervals between 20 and 100 percent of the PSC peak before and after the peak respectively. Event detection was set to 1.5 times the background noise. Electrophysiological data were analyzed in Easy Electrophysiology. Threshold was detected based on the positive peaks occurring above 10 mV/ms in the first derivative of the membrane potential. A 500 m ramp was used to analyze the rheobase, which was characterized as the lowest current inject to elicit an action potential.

After patch clamp recordings, slices were immediately fixed in 4% PFA for 2–5 h, rinsed in PBS (3 times, 30 min intervals) and kept overnight at 4°C. The same procedure as described above, was performed for immunostaining with 15’ washes.

### Morphology

Images stacks (delta 1) for morphological reconstruction were acquired using a Nikon A1 Confocal microscope (×40 objective) and the Nikon Instruments Elements software. Morphological quantification was done using the Surface and Filament tools in the IMARIS software. The automatic component of the filament tool reconstructed the dendritic field of the neuron with the dendrite beginning point set to 15 µm and the end point set to 1 µm. The volume of dendritic spread was found by using the Convex Hull Xtension of IMARIS.

### Statistics

All statistical analysis was completed using ‘Prism 8’ by GraphPad. If normally distributed, significance between data sets was examined with a parametric students t-test.

Datasets with three groups were tested using a one-way ANOVA. Datasets with two variables were tested with a two-way ANOVA with Bonferroni *post hoc* multiple comparisons. Individual data values were considered outliers if they extended beyond the mean ± 2.5x standard deviation. A critical α value of 0.05 was employed to determine significance, which is signified by *p* < 0.05*; *p* < 0.01**; *p* < 0.001***; *p* < 0.0001****. Error bars in all graphs show the standard error of the mean.

## Results

### Expression of *Cntnap2* in the mouse brain

Cntnap2/Caspr2 is known to be ubiquitously expressed throughout the brain (Poliak et al., 1999) and its absence in the *Cntnap2* KO mouse model and in humans has been shown to trigger behavioral characteristics of ASD (Penagarikano et al., 2011; Scott et al., 2017). Yet, the intricacies of *Cntnap2* expression during brain development are not well characterized.

**FIGURE 1:**
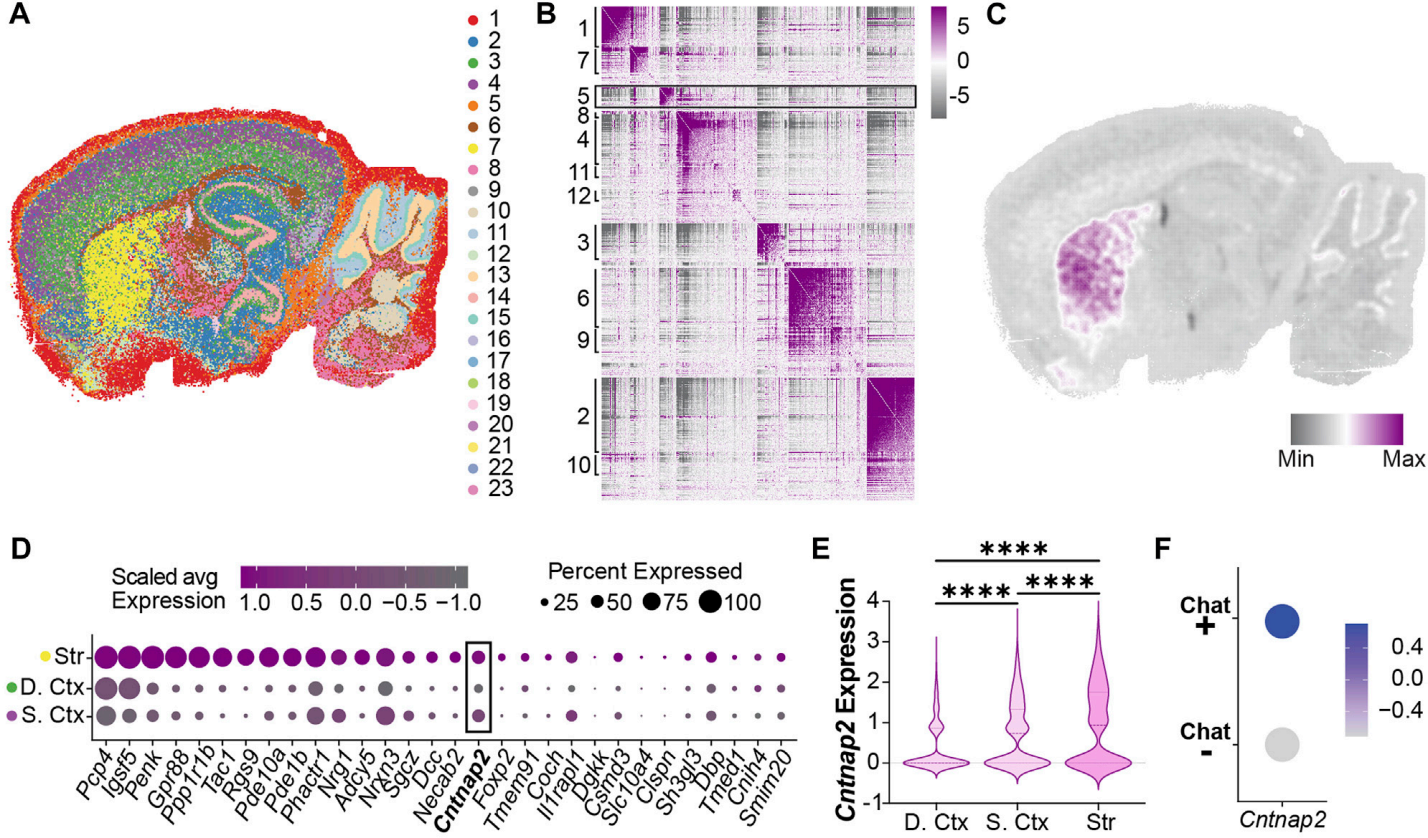
*Cntnap2* mRNA expression in the adult striatum **(A)** Unsupervised clustering in a sagittal adult mouse brain slice identified 23 clusters following stereo-seq analysis at a 50 bin resolution. **(B)** Genes with significant spatial autocorrelation (FDR <0.05) grouped into 12 distinct modules. **(C)** Module 5 genes are specifically enriched in the striatum. **(D)** Relative expression of module 5 genes in striatum and cortex clusters (Str: striatum, cluster 7, **(D)**. Ctx: deep cortex, cluster 3, S. Ctx: superficial cortex: cluster 4). **(E)** *Cntnap2* mRNA expression levels in the deep and superficial cortex, and striatum clusters. Log2 fold change and adjusted p values were calculated pairwise (deep ctx vs. sup. ctx: log2FC = 0.31, *p* < 0.0001; sup. ctx vs. str: log2FC = 0.17, *p* < 0.0001; deep ctx vs. str: log2FC = 0.48, *p* < 0.0001). **(F)** *Cntnap2* expression in two populations segregated based on Choline Acetyltransferase (ChAT, marker of cholinergic interneurons). Scale: average expression.

To examine this, we performed spatial transcriptomics (Stereo-seq) on adult brain tissue to map *Cntnap2* mRNA expression to anatomical locations. Unbiased clustering found 23 clusters that mapped onto known brain regions (Figure 1A), such as the striatum (cluster 7), deep layers of the cortex (cluster 3) and superficial layers of the cortex (cluster 4). Further clustering of spatially defined co-expressed genes generated 12 distinct modules (Figure 1B, Supplementary Figure S1A), revealing that the striatum (Figure 1C; Module 5) displays an individual genetic profile involving known biological pathways such as learning, cognition and locomotor behaviour (Supplementary Figure S1B). Furthermore, we found in this module an enriched expression of *Cntnap2* (Figure 1D; Supplementary Figure S1C). When compared to cortical regions (deep vs*.* superficial layers), the striatum showed significantly higher levels of *Cntnap2* (Figures 1D, E). Interestingly, we also found that striatal cholinergic interneurons, expressing Choline Acetyltransferase (ChAT) presented higher *Cntnap2* levels, compared to the rest of the striatal ChAT-negative population (Figure 1F).

**FIGURE 2:**
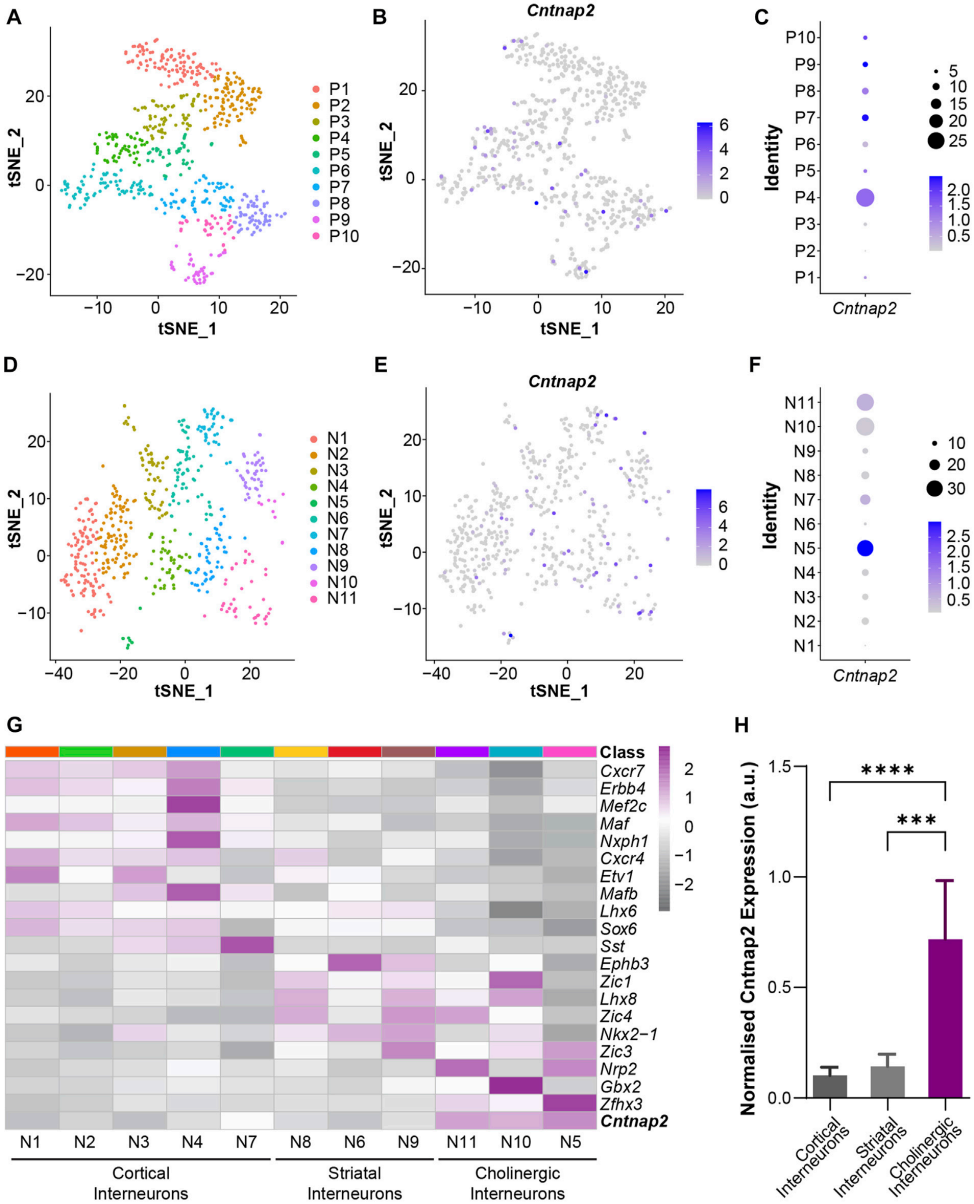
*Cntnap2* mRNA expression in the embryonic medial ganglionic eminence at E12.5 and E14.5. **(A)** t-SNE plot depicting progenitor clusters by unsupervised clustering. Progenitors from E12.5 and E14.5 MGE were jointly classified together into 10 clusters. Cells are colored by cluster identity. **(B)** t-SNE plot shows *Cntnap2* expression in MGE progenitor cells. **(C)** *Cntnap2* expression within each progenitor cell cluster. Percent expressed represented by dots (right), scale: average expression. **(D)** t-SNE plot showing neuronal clusters by unsupervised clustering. Neurons from E12.5 and E14.5 MGE were jointly classified into 11 clusters. Cells are colored by cluster identity. **(E)** t-SNE plot shows Cntnap2 expression in MGE neurons. **(F)** *Cntnap2* expression within each post-mitotic neuron cluster. Percent expressed represented by dots (right), scale: average expression. **(G)** Heatmap illustrating the expression pattern of known marker genes of cortical interneurons, striatal interneurons and cholinergic interneurons in the identified neuronal clusters. **(H)** Normalized expression of *Cntnap2* in cortical interneurons, striatal interneurons and cholinergic interneurons. (n = 355 cortical interneurons, 158 striatal interneurons, 57 cholinergic interneurons, *p* < 0.0001, one-way ANOVA; cortical interneurons vs. striatal interneurons *p* = 0.999, cortical interneurons vs. cholinergic interneurons *p* < 0.0001, striatal interneurons vs. cholinergic interneurons *p* = 0.0001, Bonferroni *post hoc* test).

To confirm whether this feature originates from brain development, we investigated *Cntnap2* expression at embryonic stages. Cntnap2/Caspr2 protein expression has been detected in the mouse brain as early as E14 (Penagarikano et al., 2011). However, its expression pattern in the embryonic ganglionic eminences is largely unknown. To this end, we used a published single-cell RNA-sequencing dataset of embryonic mouse ganglionic eminences (Mi et al., 2018) and performed new analyses on progenitors and postmitotic neurons of E12.5 and E14.5 MGE. We used unsupervised clustering to classify MGE progenitors into 10 transcriptionally distinctive clusters (P1-P10) with characteristic regional and temporal patterns (Figure 2A; Supplementary Figures S2A–F), and *Cntnap2* expression was quantified across these clusters (Figure 2B). We found that *Cntnap2* was highly expressed in some E12.5 and E14.5 clusters (e.g., P4, P7, P9, Figure 2C), corresponding to progenitor cell populations allocated primarily in the ventricular zone (VZ; P9) and subventricular zone (SVZ; P4 & P7) and of the MGE (Supplementary Figure S2F). Unsupervised clustering analysis also revealed 11 postmitotic neuron clusters (N1-N11; Figure 2D), and we observed *Cntnap2* expression in some of these clusters (Figures 2E, F). We also found that *Cntnap2* expression is particularly enriched in cell clusters annotated as cholinergic neurons, compared to cortical and striatal interneurons (Figures 2G, H). Overall, these data indicate that *Cntnap2* is expressed in a subset of progenitors and postmitotic neurons of embryonic MGE and remains enriched in the MGE-derived cholinergic interneurons of the striatum in adult. We hypothesized that this specific pattern of expression could underlie some precise alterations to striatal cells in the absence of *Cntnap2*. Specifically, we questioned whether there would be a change to MGE cell proliferation in the *Cntnap2* KO mouse model of autism, potentially contributing to ASD aetiology.

### MGE proliferation in the *Cntnap2* KO mice

**FIGURE 3:**
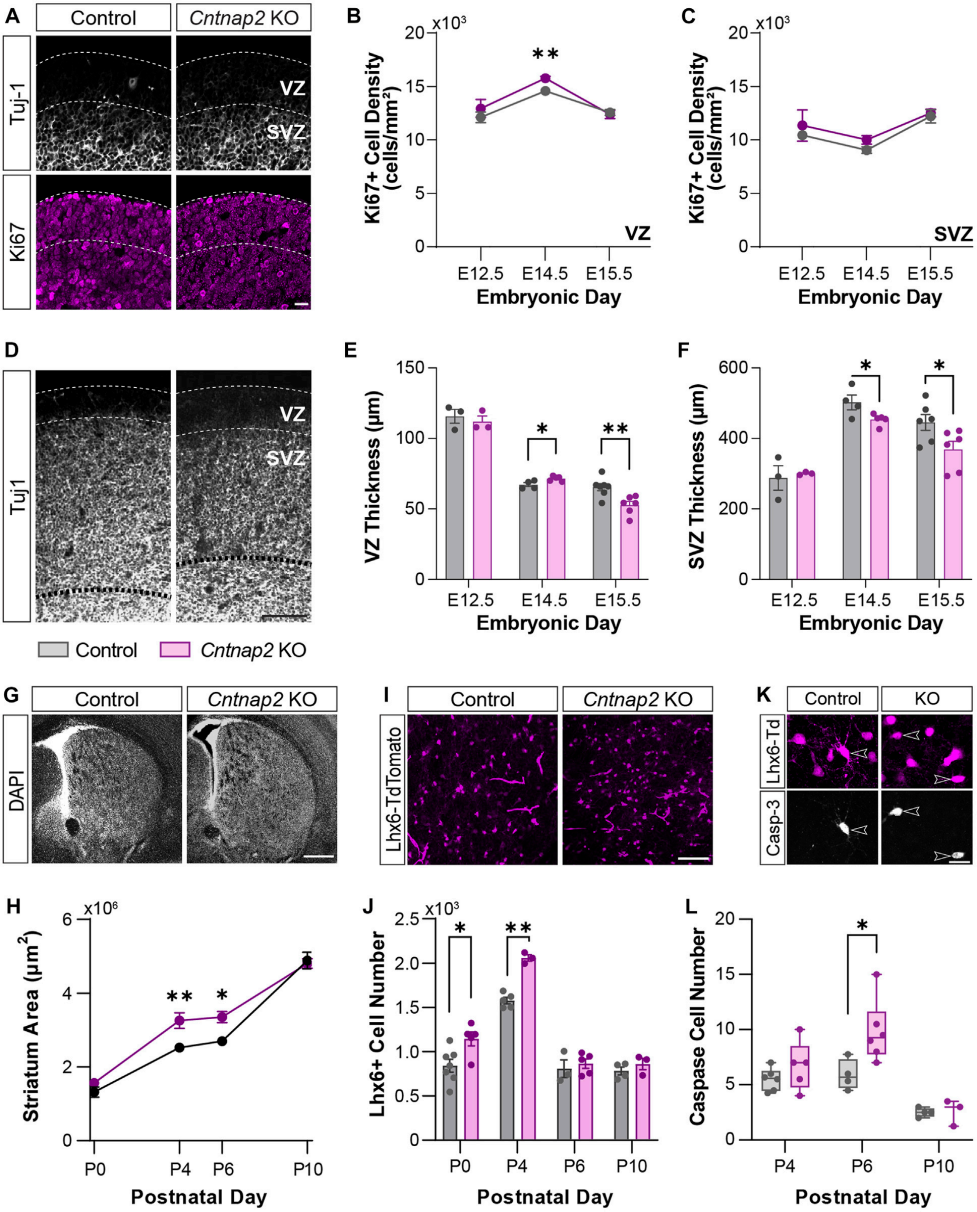
MGE-derived interneuron cell numbers at early stages of development. **(A)** Ki67-positive progenitor cells (magenta) in the VZ and SVZ of the MGE delineated by Tuj-1 staining (white), in Control and the *Cntnap2* knockout, scale: 50 µm. **(B)** Ki67-positive cell density in the VZ (E12.5: n = 3 Control, 3 KO, *p* = 0.440; E14.5: n = 4 Control, 5 KO, *p* = 0.001; E15.5: n = 6 Control, 5 KO, *p* = 0.718) and **(C)** in the SVZ of the MGE across embryonic stages (E12.5: n = 3 Control, 3 KO, *p* = 0.578; E14.5: n = 4 Control, 5 KO, *p* = 0.106; E15.5: n = 6 Control, 5 KO, *p* = 0.686). **(D)** Thickness of the VZ and SVZ in the MGE, shown by Tuj-1 staining, scale: 100 µm. **(E)** Quantification of the thickness of the VZ (E12.5: n = 3 Control, 3 KO, *p* = 0.584; E14.5: n = 4 Control, 5 KO, *p* = 0.027; E15.5: n = 7 Control, 6 KO, *p* = 0.005) and **(F)** SVZ (E12.5: n = 3 Control, 3 KO, *p* = 0.739; E14.5: n = 4 Control, 5 KO, *p* = 0.046; E15.5: n = 6 Control, 6 KO, *p* = 0.040) across embryonic stages. **(G)** DAPI staining of the striatum in Control and *Cntnap2* KO, scale: 500 µm. **(H)** Quantification of the striatum area across early postnatal stages in Control and *Cntnap2* KO (P0: n = 8 Control, 5 KO, *p* = 0.195; P4: n = 5 Control, 3 KO, *p* = 0.007; P6: n = 3 Control, 5 KO, *p* = 0.027; P10: n = 4 Control, 4 KO, *p* = 0.768). **(I)** Lhx6 positive interneurons in the striatum, scale: 100 µm. **(J)** The number of Lhx6 positive interneurons in the Control and *Cntnap2* KO striatum across early postnatal stages (P0: n = 7 Control, 5 KO, *p* = 0.019; P4: n = 5 Control, 3 KO, *p* = 0.0001; P6: n = 3 Control, 5 KO, *p* = 0.606; P10: n = 4 Control, 3 KO, *p* = 0.343). **(K)** Expression of the cell death marker Caspase-3 in striatal Lhx6 positive interneurons, scale: 20 µm. **(L)** Quantification of Lhx6 positive interneurons expressing Caspase-3 across early postnatal stages in the Control and *Cntnap2* KO conditions (P4: n = 6 Control, 5 KO, *p* = 0.259; P6: n = 4 Control, 5 KO, *p* = 0.033; P10: n = 4 Control, 3 KO, *p* = 0.898). Unless otherwise specified, a Student’s t-test was used to determine significance, *p* < 0.05:*, *p* < 0.01:**, *p* < 0.001:***, *p* < 0.0001:****.

We examined the density of the proliferation marker Ki67 in the MGE, at “early” (E12.5) and “late” (E14.5 & E15.5) embryonic stages, in the control and *Cntnap2* KO mice (Figure 3A). We observed that the number of progenitor cells in the VZ proliferative region were transiently increased at E14.5, with no changes at E12.5 or E15.5 (Figure 3B) and in the SVZ (Figure 3C). To confirm this was not a result of cells remaining in the cell cycle and/or accumulating in the VZ, we labelled cells with EdU 1 h prior to immunostaining (Flomerfelt and Gress, 2016). We observed no changes to the density or proportion of EdU^+^ cells expressing Ki67 (i.e., cells that were in DNA synthesis (S) phase an hour prior; Supplementary Figures S3A–E). During the late stages of MGE development (>E14), cell-division is biased towards neurogenic divisions and is correlated with a reduction in the size of the VZ and SVZ progenitor pools (Turrero Garcia and Harwell, 2017). We wondered whether this process was altered in the *Cntnap2* KO condition, as alterations in progenitor cells has been reported in ASD (Satterstrom et al., 2020). Whilst no changes were seen in the VZ and SVZ size at E12.5, we found that the thickness of the VZ was significantly larger at E14.5, and sharply decreased by E15.5 (Figures 3D, E). On the other hand, the SVZ remained smaller at both E14.5 and E15.5 (Figures 3D, F). Together, this indicates a short period of enhanced VZ proliferation around E14.5, which is coupled to a larger VZ size and higher density of progenitor cells. This is followed by a rapid change to a more “mature” state of the VZ and SVZ, which decrease in size earlier than in control conditions at E15.5. Interestingly, we found that this was associated with a large increase in the number of interneurons migrating towards the cortex (Supplementary Figures S3F–H **)**. We next wanted to assess whether the enhanced proliferation and migration contribute to changes in the early postnatal brain.

### MGE-derived interneuron cell number in the Cntnap2 KO mice at perinatal stages

Previous studies have shown either no change or a decrease to GABAergic and cholinergic interneuron populations in adulthood (Penagarikano et al., 2011; Lauber et al., 2018). To reconcile this with the embryonic increase in proliferation and migration we observed, we first quantified the morphological differences in the early postnatal mouse brain. Indeed, enlargement in total brain volume have been reported in ASD patients (Sacco et al., 2015). Yet, such deficits were not studied in the *Cntnap2* KO mice. Here, we found that the striatum size is enlarged in the *Cntnap2* KO condition, compared to control, specifically at P4 and P6, then rectified by P10 (Figures 3G, H). This was associated with a marked increase in the absolute number of striatal interneurons at early postnatal stages, labelled with the specific marker of MGE-derived precursors and interneurons, LIM homeobox 6 (Lhx6) transcription factor (Liodis et al., 2007); Figures 3I, J) between P0 and P4. However, striatal Lhx6 interneuron number is normalized from P6 in the *Cntnap2* KO mice (Supplementary Figure S3I), and no changes in cortical Lhx6^+^ cell density and prefrontal cortex thickness was observed (Supplementary Figures S3J–L).

Since the first postnatal week corresponds to a critical period for brain development, with notable cell death episodes (Wong et al., 2018; Sreenivasan et al., 2022), we hypothesized that changes to apoptosis were responsible for the restoration of cell number. As previously described in the striatum, Lhx6^+^ cell death occurs around P6 (Sreenivasan et al., 2022). Quantification of the marker Caspase-3 across early postnatal stages within the Lhx6+ interneuron population showed a 49% cell loss at P6 in control conditions, as previously reported (Sreenivasan et al., 2022), and an increase to 58% cell loss in the *Cntnap2* KO mice (Figures 3K, L). More cell death was also seen in the neocortex in the *Cntnap2* KO mice, compared to controls (data not shown). Together, these findings show that in the absence of *Cntnap2*, an embryonic increase in cell proliferation in the MGE leads to an increase in interneuron numbers at early postnatal stages, which is compensated for by additional cell death to rectify this back to control levels. As previously described (Vogt et al., 2017), we did not find any differences in the number of MGE-derived cells in the adult striatum (n = 3 control and n = 3 KO; data not shown). However, alterations to cellular development during embryonic and early postnatal periods are likely to trigger morpho-functional modifications that could contribute to ASD aetiology. Therefore, we next analyzed whether the properties of the striatal Lhx6^+^ interneurons where modified at these stages.

### Morpho-functional properties of the MGE-derived striatal interneurons in the *Cntnap2* KO mice

**FIGURE 4:**
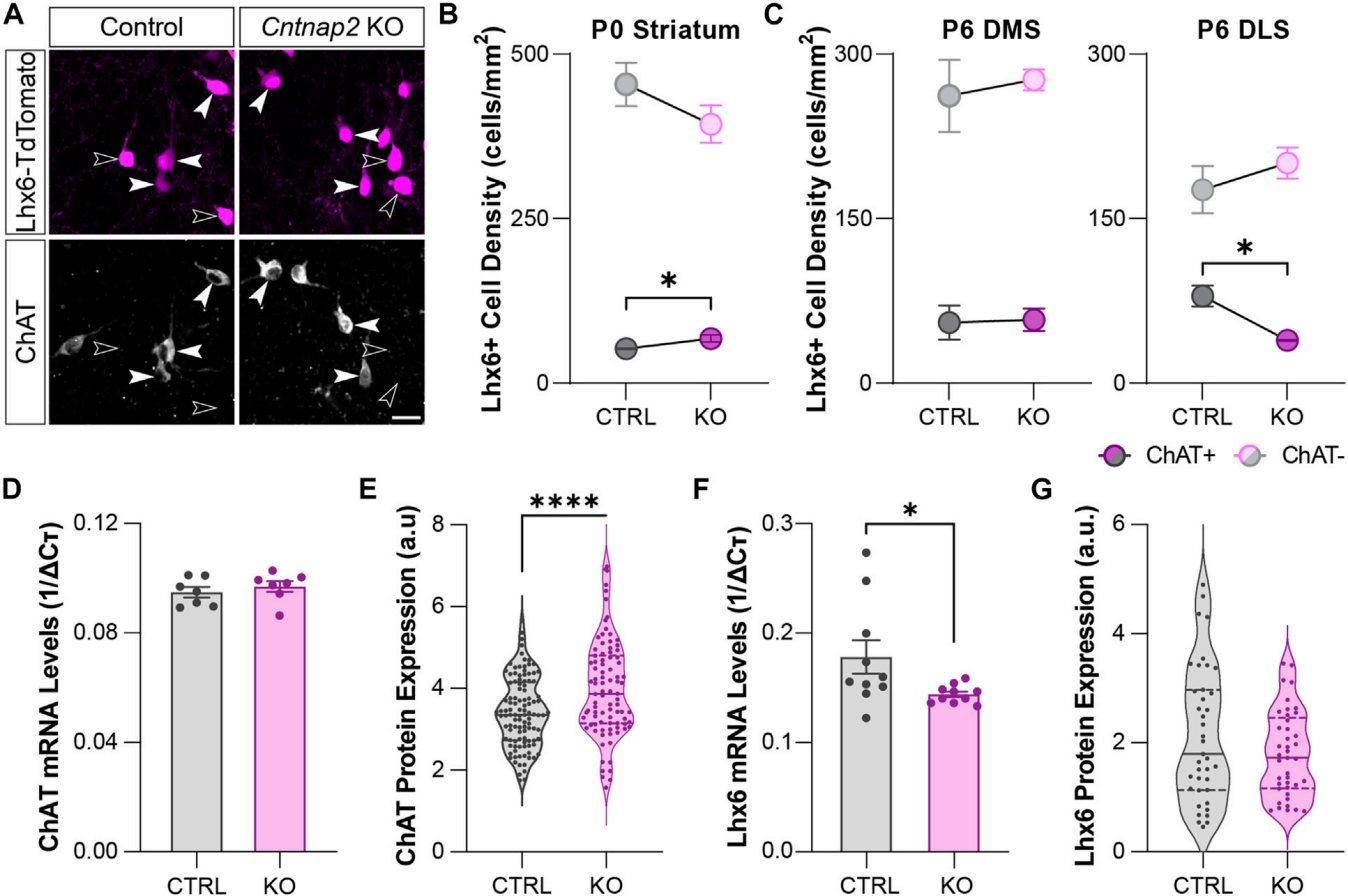
Molecular identity of the developing striatal Lhx6+ interneurons in Control and *Cntnap2* KO mice **(A)** Expression of the cholinergic marker ChAT in the Lhx6+ interneurons of the striatum at P6, scale: 20 µm. **(B)** Density of ChAT positive and ChAT negative Lhx6+ interneurons in the Control and *Cntnap2* KO striatum at P0 (n = 5 Control, 3 KO, *p* = 0.043 ChAT+, *p* = 0.259 ChAT-). **(C)** Density of ChAT positive and ChAT negative Lhx6+ interneurons in the Control and *Cntnap2* KO at P6, in the dorsomedial (DMS, n = 3 Control, 4 KO, *p* = 0.893 ChAT+, *p* = 0.644 ChAT-) and dorsolateral (DLS, n = 3 Control, 3 KO, *p* = 0.016 ChAT+, *p* = 0.398 ChAT-) striatum. **(D)** Relative *ChAT* mRNA expression in the striatum in Control and *Cntnap2* KO conditions at P6 (n = 7 Control, 7 KO, *p* = 0.465). **(E)** Normalized ChAT protein levels in striatal cholinergic interneurons, in Control and *Cntnap2* KO conditions (n = 110 cells, 4 Control, 91 cells, 3 KO, *p* = 0.00005). **(F)** Relative *Lhx6* mRNA expression in the striatum, in Control and *Cntnap2* KO conditions at P6 (n = 10 Control, 10 KO, *p* = 0.041). **(G)** Normalized Lhx6 protein levels in striatal interneurons, in Control and *Cntnap2* KO conditions (n = 40 cells, 4 Control, 42 cells, 3 KO, *p* = 0.129). Unless otherwise specified, a Student’s t-test was used to determine significance, *p* < 0.05:*, *p* < 0.01:**, *p* < 0.001:***, *p* < 0.0001:****.

Analysis of spatial transcriptomics and single-cell RNA sequencing data indicated higher levels of *Cntnap2* in putative MGE-derived cholinergic neuron populations (Figure 1 and Figure 2), potentially suggesting that Cntnap2 plays a specific role in CINs. To determine whether this population was preferentially altered following *Cntnap2* deletion, and likely contribute to the ASD-related alterations, we quantified the proportion of the Lhx6^+^ interneurons expressing ChAT at early postnatal stages. At P0, there was a greater proportion of interneurons that were cholinergic in the *Cntnap2* KO condition (Figures 4A, B) and correspondingly, this proportion was decreased at P6, specifically in the dorsolateral striatum (DLS; Figure 4C). This decrease was specific to the Lhx6^+^ cholinergic cell population, as we did not find any alterations in the cholinergic Lhx6-negative cells in the striatum (Supplementary Figures S4A, B).

We next wondered whether the molecular profile of these cells was changed. We first analyzed the molecular profile of Lhx6-expressing interneurons by qRT-PCR, at embryonic and perinatal stages in the control and *Cntnap2* KO mice. Whilst we found that expression levels of genes related to cholinergic identity such as *ChAT*, *Lhx8*, *Gbx2* and *Zic4* (Ahmed et al., 2019) remain unchanged between the two conditions in the MGE and the postnatal striatum (P6: Figure 4D and Supplementary Figure S4C), we observed increased levels of ChAT protein within striatal cholinergic Lhx6^+^ interneurons at P6 (Figure 4E), but not cholinergic Lhx6-negative interneurons (Supplementary Figure S4D). We also quantified a decrease in *Lhx6* mRNA levels (Figure 4F), with no change to the Lhx6 protein expression levels in the Lhx6^+^ cells at P6 (Figure 4G). This data suggests different post-transcriptional control of the striatal Lhx6^+^ interneurons in the *Cntnap2* KO mice, compared to controls.

**FIGURE 5:**
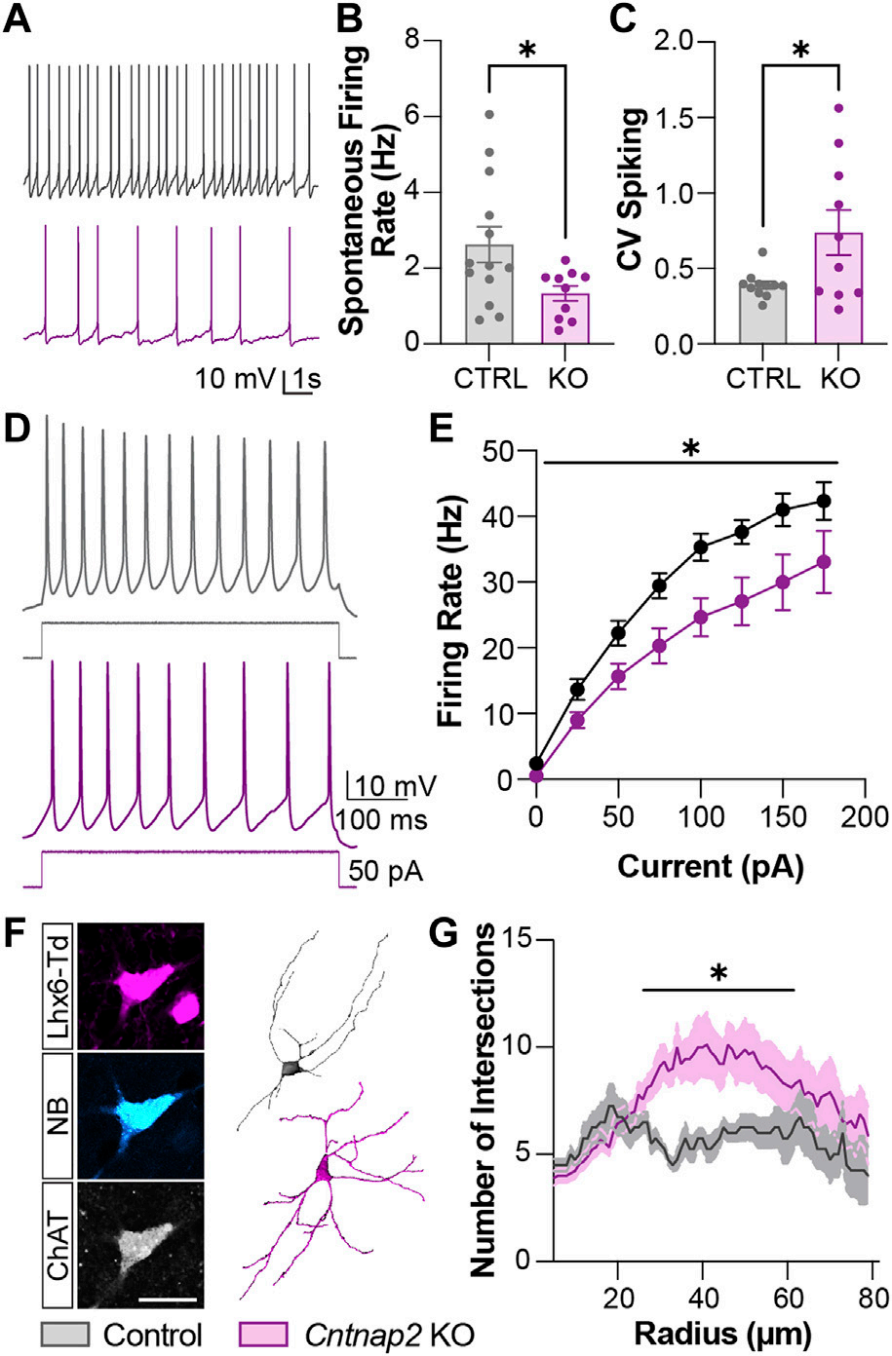
Morpho-functional alterations to the Lhx6+ cholinergic interneuron population in the *Cntnap2* KO mice. **(A)** Spontaneous tonic firing of the P6 Lhx6+ cholinergic interneurons at resting membrane potential. **(B)** Quantification of cholinergic interneuron tonic firing rate firing in Control and *Cntnap2* KO conditions (n = 13 Control, 10 KO cells; *p* = 0.034). **(C)** Coefficient of spike variation of cholinergic interneuron tonic firing in Control and *Cntnap2* KO conditions (n = 11 Control, 10 KO cells; *p* = 0.026). **(D)** Evoked firing of P6 Lhx6+ cholinergic interneurons. **(E)** Evoked firing rate in Control and *Cntnap2* KO conditions across increasing current inputs (n = 8 Control, 12 KO, p_genotype_ = 0.015, p_interaction_ = 0.025, two-way ANOVA, repeated measures). **(F)** Patched cholinergic interneuron loaded with neurobiotin (left) used for morphological reconstruction (right) in Control and *Cntnap2* KO conditions, scale: 20 µm. **(G)** Sholl analysis measuring the number of dendrites intersecting circles increasing in radius by 1 µm (n = 4 Control, 9 KO, *p* = 0.049, two-way ANOVA). Unless otherwise specified, a Student’s t-test was used to determine significance, *p* < 0.05:*, *p* < 0.01:**, *p* < 0.001:***, *p* < 0.0001:****.

We then performed *in vitro* whole-cell patch clamp recording of the Lhx6^+^ cells in the P6 striatum, to assess whether the electrical and morphological features of those cells were altered in the absence of *Cntnap2*. We separated Lhx6^+^ CINs and GABAergic neurons in the control and *Cntnap2* KO mice, based on their firing properties and *post hoc* staining (Supplementary Figure S5A; Tables 1, 2). The Lhx6^+^ CINs display spontaneous tonic activity in current-clamp mode (Ahmed et al., 2019) which appears reduced (Figures 5A, B) and far more irregular in the *Cntnap2* KO condition (Figure 5C). This was also reflected in evoked firing rates upon current injection, which were also significantly decreased (Figures 5D, E), whilst the input resistance was lower (Table 1). Sag ratio and spike adaptation were similar in control and KO Lhx6^+^ CINs (Table 1), suggesting similar level of maturation of the conductances. Reduced firing frequency was also observed in the adult CINs, suggesting persistent developmental alterations in the *Cntnap2* KO conditions (Reynolds et al., unpublished).

**TABLE 1:** Intrinsic properties of Lhx6+ striatal cholinergic interneurons in the control and *Cntnap2* KO mice at P6.

Lhx6^+^ cholinergic interneurons					
Property	Control		KO		*p*
	Mean	n	Mean	n	
Resting membrane potential (mV)	−44.90 ± 1.55	16	−47.44 ± 2.27	12	0.368
Input resistance (MΩ)	551.24 ± 60.17	14	408.61 ± 23.79	12	0.049
Membrane capacitance (pF)	596.63 ± 59.82	14	802.39 ± 125.74	12	0.135
Sag ratio	0.27 ± 0.03	10	0.30 ± 0.07	7	0.676
Spike adaptation index	0.60 ± 0.05	14	0.60 ± 0.05	11	0.926
Latency to first spike (ms)	19.64 ± 3.13	14	21.59 ± 3.22	11	0.671
Action potential threshold (mV)	−35.59 ± 1.18	15	−36.58 ± 1.97	12	0.655
Action potential amplitude (mV)	51.95 ± 2.13	15	50.13 ± 3.62	12	0.654
Action potential rise time (ms)	1.07 ± 0.09	15	0.77 ± 0.06	12	0.017
Action potential decay (ms)	2.05 ± 0.01	15	2.02 ± 0.02	12	0.229
Action potential halfwidth (ms)	2.71 ± 0.16	15	2.50 ± 0.14	12	0.347
Action potential fast AHP (mV)	9.02 ± 1.23	15	6.11 ± 1.35	12	0.126
Action potential medium AHP (mV)	−13.77 ± 1.22	15	−13.78 ± 1.00	12	0.993

AHP: Afterhyperpolarization. Statistics from two-sample Student’s t-test.

**TABLE 2:** Intrinsic properties of Lhx6+ striatal GABAergic fast-spiking interneurons in the control and *Cntnap2* KO mice at P6.

Lhx6^+^ putative fast spiking interneurons					
Property	Control		KO		*p*
	Mean	n	Mean	n	
Resting membrane potential (mV)	−54.45 ± 1.50	22	−53.98 ± 3.09	13	0.880
Input resistance (MΩ)	780.28 ± 75.02	19	406.55 ± 47.03	14	0.0005
Membrane capacitance (pF)	805.33 ± 126.76	19	1064.36 ± 273.87	14	0.357
Latency to first spike (ms)	270.01 ± 35.37	6	315.87 ± 25.58	10	0.303
Action potential threshold (mV)	−35.49 ± 1.43	19	−32.45 ± 1.91	11	0.184
Action potential amplitude (mV)	48.15 ± 1.80	19	44.59 ± 3.43	11	0.320
Action potential rise time (ms)	0.72 ± 0.05	19	0.59 ± 0.05	11	0.078
Action potential decay (ms)	1.80 ± 0.05	19	1.59 ± 0.09	11	0.031
Action potential halfwidth (ms)	1.74 ± 0.12	19	1.22 ± 0.11	11	0.006
Action potential fast AHP (mV)	-2.69 ± 1.72	19	-10.89 ± 1.09	11	0.002
Action potential medium AHP (mV)	−13.15 ± 0.92	19	−15.37 ± 0.70	11	0.105

AHP: Afterhyperpolarization. Statistics from two-sample Student’s t-test.

Because cell activity regulates morphological development and Cntnap2 has been reported to stabilize interneuron dendritic arbors (Gao et al., 2018), we next investigated whether cell morphology was affected in the *Cntnap2* KO condition. We performed morphological analysis of the striatal Lhx6^+^ CINs to verify whether their development at P6 was impaired in the absence of *Cntnap2* (Figure 5F). We found that Lhx6+ CINs were more complex at proximal dendrites (*i.e.* ∼ 50 µm from the soma; Figure 5G), and presented increased branching and dendritic segments (Supplementary Figures S4E–H).

We also checked the electrophysiological properties of the putative Lhx6+ GABAergic interneurons (that are negative for ChAT immunostaining; Supplementary Figure S5A). Whilst most of the basic intrinsic properties remained unchanged (Table 2), we found a significant increase in the rheobase (Supplementary Figure S5B), associated with a lower firing at intermediate depolarizing steps, but not at maximum firing (Supplementary Figures S5C, D). The morphological characteristics were also changed, with more complex arborization at distal dendrites (i.e. >50 µm from the soma) and larger volume of spread (Supplementary Figures S5E, J).

To further characterize the changes in synaptic connectivity in the *Cntnap2* KO mice, we performed voltage-clamp recordings of the inhibitory and excitatory inputs onto Lhx6+ interneurons at P6. We found that cells receive similar GABAergic and α-Amino-3-hydroxy-5-methyl-4-isoxazolepropionic acid (AMPA)/Kainate-driven glutamate inputs (Supplementary Figures S6A–J) in control and *Cntnap2* KO mice. However, Lhx6+ interneurons receive more N-methyl-D-aspartate (NMDA) receptor-driven glutamate inputs at P6 in the KO condition (Supplementary Figures S6K–O). This was associated with a shift in the expression of glutamate receptors such as the metabotropic receptor mgluR5 (Supplementary Figures S6P, Q), and glutamate receptor NR2 C & D subunits (Supplementary Figures S6R, S), but not NR1 and NR2 A & B subunits (data not shown). Overall, these results reveal lower cell excitability and altered synaptic connectivity in the Lhx6^+^ interneuron population of the P6 striatum in the *Cntnap2* KO mice.

## Discussion

From early prenatal stages through childhood, the striatum undergoes an avalanche of developmental events that shape network formation (Dehorter et al., 2012; Dehorter and Del Pino, 2020). Yet, how the maturation of the striatal interneurons is sculpted in ASD has remained poorly investigated. Here, we demonstrated that the developmental trajectory of striatal MGE-derived interneurons is perturbed in the *Cntnap2* KO mouse model of ASD from embryonic stages. In particular, we uncovered enhanced proliferation of neural progenitor cells, followed by an early maturation of the VZ and SVZ of the MGE, and accumulation of cells in the migratory stream. This led to transitory changes to the developing brain size, and heightened postnatal cell loss (20% increase) during the canonical window of developmental cell death in the striatum [around P6; (Sreenivasan et al., 2022)]. These alterations were associated with intrinsic perturbations to interneuron physiological properties, neuronal differentiation and connectivity that are likely to support striatal-related impairments described in the *Cntnap2* KO mice model (Scott et al., 2017).

### Altered proliferation and migration of the striatal cholinergic interneurons following *Cntnap2* deletion

Using high-resolution spatial transcriptomics (Stereo-seq) and scRNAseq data from the MGE, we described the spatial pattern of expression of *Cntnap2* in the adult brain and at embryonic stages in the MGE, where we could detect some *Cntnap2* ^+^ cells, as early as E12. We reveal that *Cntnap2* expression is enriched in the progenitor cells, as well as post-mitotic neurons of the striatum and in particular, in the MGE-derived cholinergic interneurons. Since we observed a shift in the proportion of Lhx6^+^ CINs produced, and enhanced cell-specific cell death in the *Cntnap2* KO mice, we established that deletion of *Cntnap2* preferentially affected these cells. Considering Cntnap2 is known to control processes that occur in more developed cells (e.g., myelination, synapse maintenance…), it was interesting to find *Cntnap2* in progenitor cells. Does Cntnap2 regulate neural progenitor cell physiology? Some of our data from *in vitro* patch clamp recordings at E16 suggest that the basic electrical properties of the progenitor cells are unchanged in the *Cntnap2* KO condition (Data not shown). However, the Cntnap2 protein expression levels and precise subcellular distribution of the potassium channel subunits Kv1.2 (Poliak et al., 1999; Scott et al., 2017) remain to be determined, to fully appreciate the functional impact Cntnap2 has on different embryonic cell types. Since *Cntnap2* is expressed ubiquitously in the developing brain, it will be necessary to use a conditional mutant to better analyze the specific intricacies of Cntnap2 that may affect cell function and early cell communication. Overall, our results emphasize the importance of further study how a human disease risk gene such as *Cntnap2* regulates interneuron development. Indeed, *Cntnap2* is expressed in the MGE progenitors cells in humans (Satterstrom et al., 2020), and in future sub-pallial cholinergic and GABAergic neurons (Shi et al., 2021), suggesting that potential therapeutic interventions could be conducted from embryonic stages to rectify the altered developmental trajectory in ASD, but also a range of neurodevelopmental disorders such as schizophrenia, intellectual disability, dyslexia, epilepsy and attention-deficit hyperactivity disorder (Rodenas-Cuadrado et al., 2014; Poot, 2015).

### Readjustment of normal cell number during the first postnatal week

Disruption of cholinergic interneurons (CINs) in the striatum generate network and behavioral modifications significant to ASD (Rapanelli et al., 2017a; Caubit et al., 2022). Yet, how developing CINs are impacted in ASD has been poorly explored. By uncovering preferential and specific developmental modifications of the striatal interneuron maturation at embryonic and early postnatal stages, we demonstrate that the MGE-derived Lhx6^+^ CINs, which represent at least 50% of the striatal cholinergic contingent (Magno et al., 2017), are primarily affected by the absence of *Cntnap2*, suggesting that these cells substantially contribute to the ASD-related alterations observed in the *Cntnap2* KO conditions. Whether this is the case for ASD in general will need to be further assessed by examining the alterations of MGE-derived cholinergic interneurons in other ASD mouse models. In addition, investigating the specific role of *Cntnap2* in CINs vs*.* other striatal cells with conditional mutants will be essential to delineate the specific role of CINs in ASD pathophysiology.

Evidence shows *in utero* alterations in ASD (Satterstrom et al., 2020), with enlargement of the brain size and cell number (Bonnet-Brilhault et al., 2018). Concurrently, we found an expansion of the MGE proliferative zone around E14.5, and a shift in Lhx6^+^ cell migration, exemplified by an early reduction of the VZ proliferative zone in the MGE, and enlarged cortical migratory stream. However, there is still no rationale about whether alterations in the scaffold protein Cntnap2 can lead to migration abnormalities in these interneurons (Rapanelli et al., 2017b). Striatal cell migration is tightly regulated (Villar-Cervino et al., 2015), and genetic and environmental factors that perturb cell migration and distribution result in improper cell positioning, and E/I imbalances in ASD (Guo and Anton, 2014; de la Torre-Ubieta et al., 2016). There are reciprocal mechanisms at play in the developing brain, whereby interneuron migration influences principal cell generation (Silva et al., 2018), and activity of the principal cells affects interneuron cell survival (Wong et al., 2018). In addition, it has been shown that cell-intrinsic properties of interneuron migration controls the flow of interneurons invading the cortex and also impairs the generation of age-matched projection neurons of the upper cortical layers (Silva et al., 2018). Indeed, previous studies have shown a redirection of late-born cortical interneurons from upper layers to deeper layers in the *Cntnap2* knockout (Penagarikano et al., 2011). This is reflected in our findings with the increase in thickness of the superficial migratory stream which underlies the increase in Lhx6^+^ cell density in the deep layers of the prefrontal cortex at early developmental stages. Cell numbers in the PFC at later developmental stages were unchanged, consistent with recent studies (Scott et al., 2017; Lauber et al., 2018).

Further study is required to decipher the mechanisms underlying the involvement of specific striatal CINs, which are known to support the pathophysiology of ASD (Caubit et al., 2022), and how changes in cell number and positioning-although transitory-influence other striatal interneurons and output neurons, either directly or by compensatory mechanisms, and the establishment of functional neuronal assemblies within the striatum. Indeed, any alterations in developing interneuron function is expected to alter the excitation-inhibition balance and, overall striatal activity, to ultimately impact activity-dependent mechanisms such as perinatal network oscillations that are fundamental to build functional circuitries (Dehorter et al., 2012). A reduction in inhibitory power in different neuronal circuits (e.g., striatum vs. cortex) is expected to have distinctive impact on the excitation-inhibition balance. For example, there are no glutamatergic neurons in the striatum, compared to the cortex. This could explain why morphological alterations in the cortical GABAergic interneurons (Gao et al., 2018) are different compared to the striatal GABAergic interneurons (Supplementary Figure S5). In terms of a mechanism, a possibility is that interneurons could adjust their polarity in response to region-specific chemotactic cues (Rodenas-Cuadrado et al., 2014) to populate their final location, affecting cell morphological architecture. However, we also cannot discard that migration of the striatal output neurons, also affected in the *Cntnap2* KO condition, could directly participate to this phenotype. Employing a conditional knockout for *Cntnap2* will address these questions and tease apart the cellular and brain region specificity of the impact of Cntnap2 in developing cell migration and connectivity.

During the first two postnatal weeks, massive cell death event [i.e., up to 50% cell death; (Wong et al., 2018)] occurs in the brain. During this key period, inactive MGE-derived interneurons are more likely to die than active neurons (Denaxa et al., 2018; Wong et al., 2018). With the marked reduction of striatal cell number and increase in caspase-positive cells detected between P4 and P6, our data reveals that apoptosis is enhanced in the MGE-derived cells of the *Cntnap2* KO mice. Whilst the canonical window of developmental cell death does not appear to be shifted in this condition, the extent of cell loss appeared increased. Interestingly, the final number of CINs is regulated by the activity of the striatal output neurons, also called spiny projection neurons (SPN). When the SPN activity is increased, CIN survival is diminished (Sreenivasan et al., 2022). Therefore, we speculate that loss of striatal CIN in the *Cntnap2* KO mice may be due to an increase in SPN activity, probably driven by an early maturation of the cortico-striatal pathway, reported in ASD mouse models (Peixoto et al., 2016). Future directions will require to study the mechanisms supporting this process, such as the involvement of molecules such as the phosphatase and tensin homolog deleted on chromosome 10 (PTEN), which controls cell death (Wong et al., 2018), and the role of its interactor Tau (Tai et al., 2020). Together with the substantial loss in MGE-derived interneurons in the *Cntnap2* KO mice, we found a large decrease in the excitability of the Lhx6^+^ interneurons of the striatum. We hypothesize that changes in early cell activities also provide a feedback loop that represents a homeostatic mechanism for fine-tuning the number of interneurons (Denaxa et al., 2018), and contribute to the striatal apoptotic process. Whether these mechanisms are conserved in other interneuron types coming from the pre-optic area, septal neuroepithelium (which give rise to 50% of the CINs (Magno et al., 2017)), caudal ganglionic eminence (Ahmed et al., 2019), and other proliferative regions (e.g., cortical VZ, lateral ganglionic eminence), to contribute to the overall phenotype observed in this study, will need to be further investigated in *Cntnap2* conditional mutants.

### Striatal interneuron function and network activity

Cntnap2 is known to play a role in cell firing properties, influencing latency of adult cell firing, *via* loss of the potassium channel subunit Kv1.2 (Vogt et al., 2017). Here, we unveiled key physiological cell deficits during a key perinatal period (Dehorter et al., 2012), during which activity-dependent mechanisms dynamically adjust the number of interneurons in developing circuits, and ultimately establish the correct proportions of neural connections in a structure to be functional (Wong et al., 2018). In other words, these critical early neural activities are thought to represent checkpoints that ensure the proper formation of neuronal circuits (Dehorter et al., 2012). In this study, we observed a reduction in cell excitability, associated with increased expression of the metabotropic glutamate receptor mgluR5 and a decrease in the expression of the NMDA NR2 C/D subunits in the *Cntnap2* KO mice, which mediate large synaptic currents in interneurons (von Engelhardt et al., 2015), confers different sensitivity of the receptors to ligands (Williams et al., 1993), and provide a critical source of Ca^2+^entry *via* trophic glutamate signaling (Paoletti et al., 2013), which could add to the impact on synaptogenesis and their well-described role in potentiation (i.e., LTP/LTD; (Luscher and Malenka, 2012)). In addition, NMDA receptor-dependent and voltage-gated calcium channels activation are also known trigger calcium influx that promotes cell survival (Desfeux et al., 2010). They also mediate transcriptional cascades to control gene expression during the development of MGE-derived interneurons. In particular, specific loss of NMDA receptors in the MGE has been shown to result in a downregulation of diverse transcriptional, synaptogenesis and membrane excitability regulatory programs (Mahadevan et al., 2021). In addition, we found less NMDA-driven inputs onto Lhx6^+^ interneurons and larger dendritic complexity, overall indicating an early maturation of the glutamatergic processing in these cells (Peixoto et al., 2016). We propose that these qualitative changes in striatal interneuron physiology will impact global neural activity levels and the formation of specific synaptic connections, as seen in other ASD mouse models (Cheyne et al., 2019; Bitzenhofer et al., 2021). A better understanding of the functional link between the early alterations in the developing interneurons, and the establishment of operative neuronal assemblies is required to appreciate the consequent behavioral deficits (i.e., repetitive movements) that are contingent to the malfunction of the mature striatal network in ASD (Penagarikano et al., 2011). For example, calcium imaging recordings should determine how shifting interneuron excitability shapes synapse-driven early network oscillations, such as the giant depolarizing potentials (Dehorter et al., 2011) to control the emergence of functional circuitry in the mature striatum (Dehorter et al., 2012).

The current literature lacks an understanding of the intricate alterations to interneuron development in ASD and how this could contribute autism aetiology. Together, this study forms the basis to further explore the mechanisms underlying deficits of the developing interneurons in ASD. These findings emphasize the importance of considering targeting developing interneurons to restore normal developmental brain trajectory in ASD.

## Data Availability

The datasets presented in this study can be found in online repositories. The names of the repository/repositories and accession number(s) can be found below: Gene Expression Omnibus accession number: GSE222383 and GSE109796.
