# Supplementary material for: Developmental deficits of MGE-derived interneurons in the Cntnap2 knockout mouse model of autism spectrum disorder
Source: Front Cell Dev Biol. 2023 Feb 1;11:1112062. doi: 10.3389/fcell.2023.1112062 (PMC9930104; doi:10.3389/fcell.2023.1112062)
Supplement: Supplementary file 1 [file DataSheet1.PDF]

Supplementary Material

1 Supplementary Figures

Supplementary Figure 1.

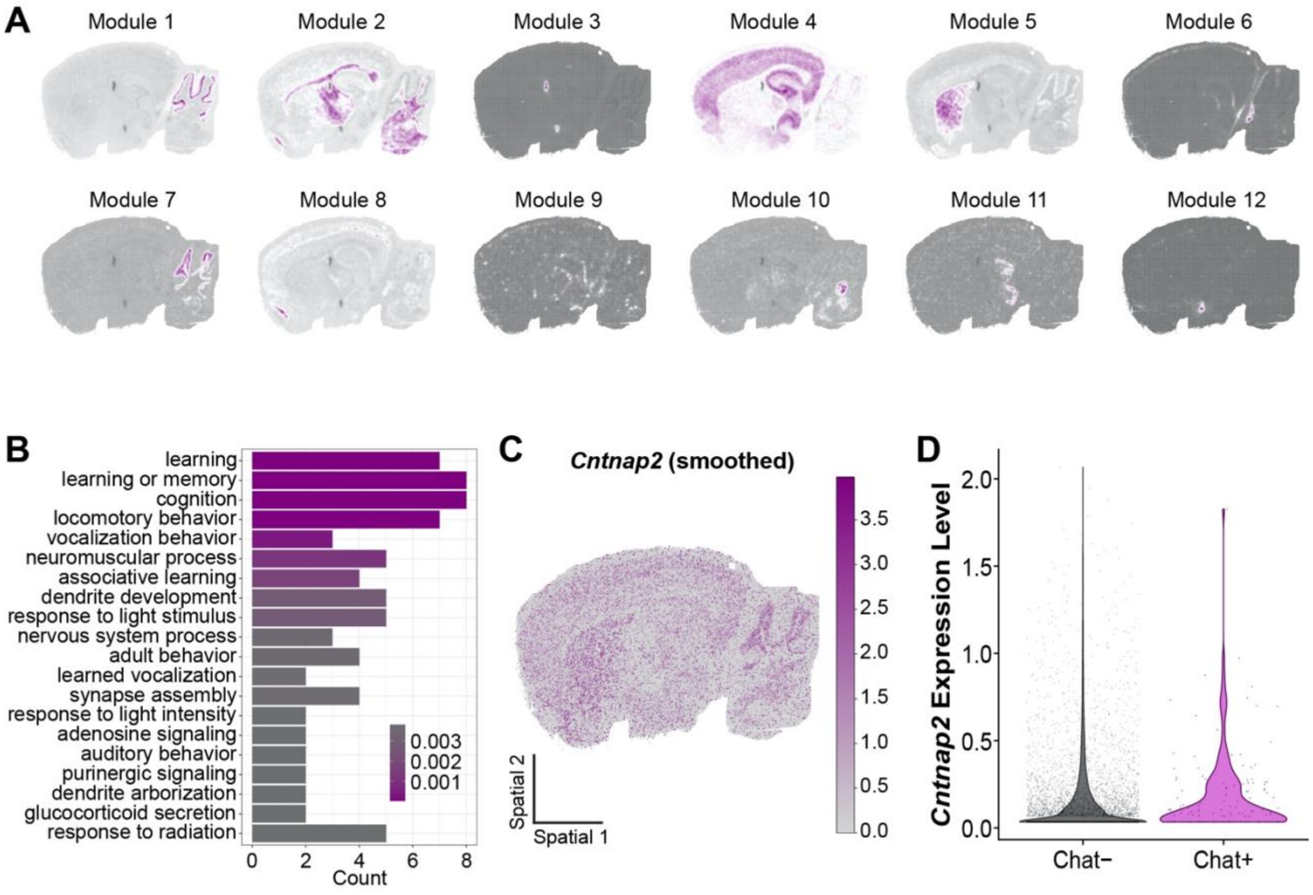

<u>Supplementary Figure 1:</u> Analysis of the Stereo-seq modules. (A) 12 distinct modules with significant spatial autocorrelation (FDR < 0.05). **(B)** Biological pathways related to genes enriched in Module 5, *i.e.* striatum. **(C)** Spatial distribution of the *Cntnap2* expression (smoothed). **(D)** Violin plots showing *Cntnap2* expression levels in cholinergic (ChAT+) and non-cholinergic (ChAT-) cell populations (n = 3927 ChAT-, 143 ChAT+, p = 0.812).

Supplementary Figure 2.

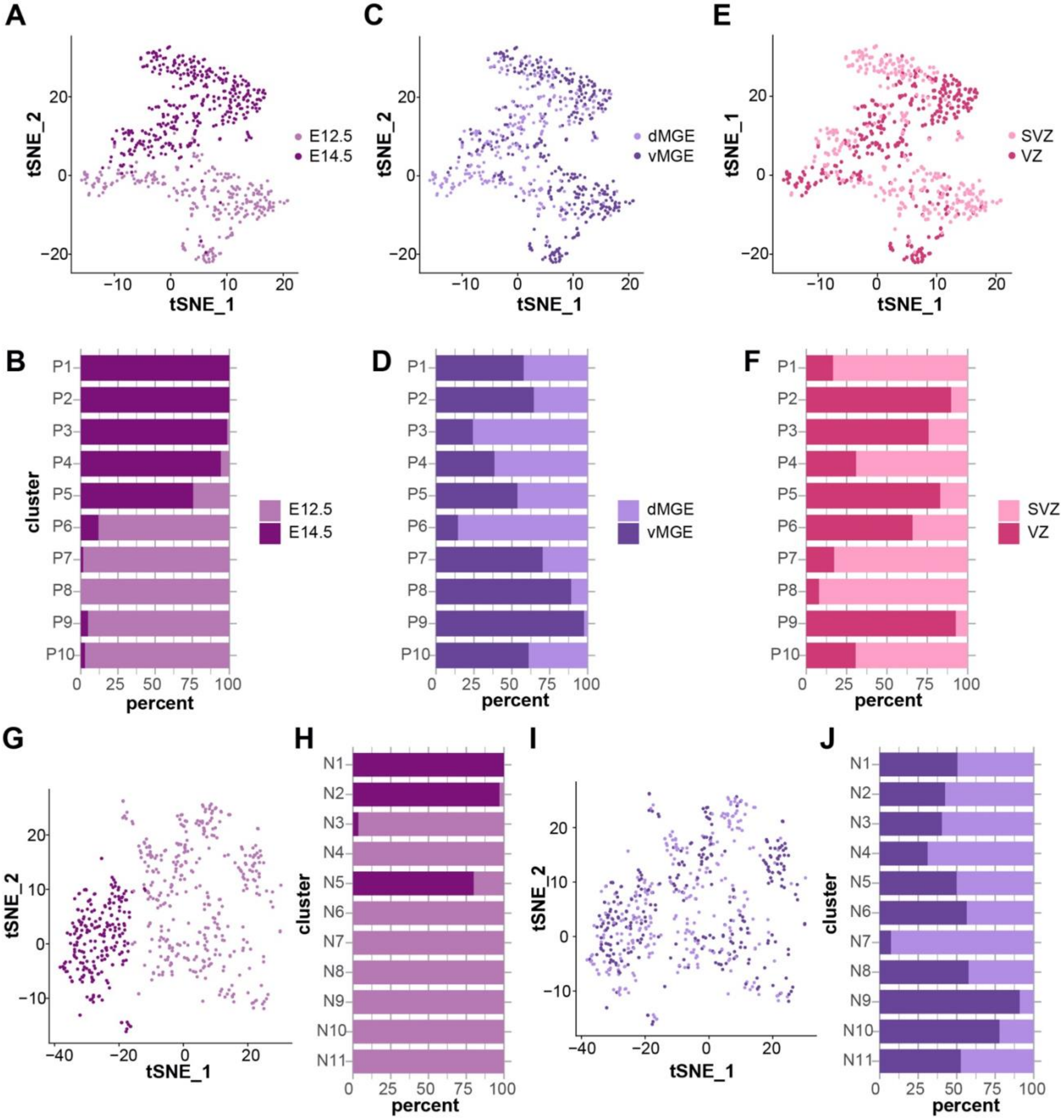

<u>Supplementary Figure 2:</u> Embryonic expression of *Cntnap2* in the MGE-derived neurons.

(A) Visualization of E12.5 and E14.5 progenitor cells by tSNE. **(B)** Relative contributions of E12.5 and E14.5 in progenitor clusters. **(C)** Visualization of the progenitor cells from different MGE subregions (dorsal: dMGE or ventral: vMGE) by tSNE. **(D)** Relative contributions of dMGE and vMGE in progenitor clusters. **(E)** Visualization of ventricular zone (VZ) and subventricular zone (SVZ) progenitor cells by tSNE. **(F)** Relative contribution of the VZ/SVZ in progenitor clusters.

(G) Visualization of E12.5 and E14.5 neurons by tSNE. **(H)** Relative contributions of each time point in neuronal clusters. **(I)** Visualization of neurons from different MGE subregions: dMGE or vMGE by tSNE. **(J)** Relative contributions of dMGE and vMGE in neuronal clusters. Unless otherwise specified, a Student's t-test was used to determine significance, $p<0.05$:*, $p<0.01$:**, $p<0.001$:***, $p<0.0001$:****.

Supplementary Figure 3.

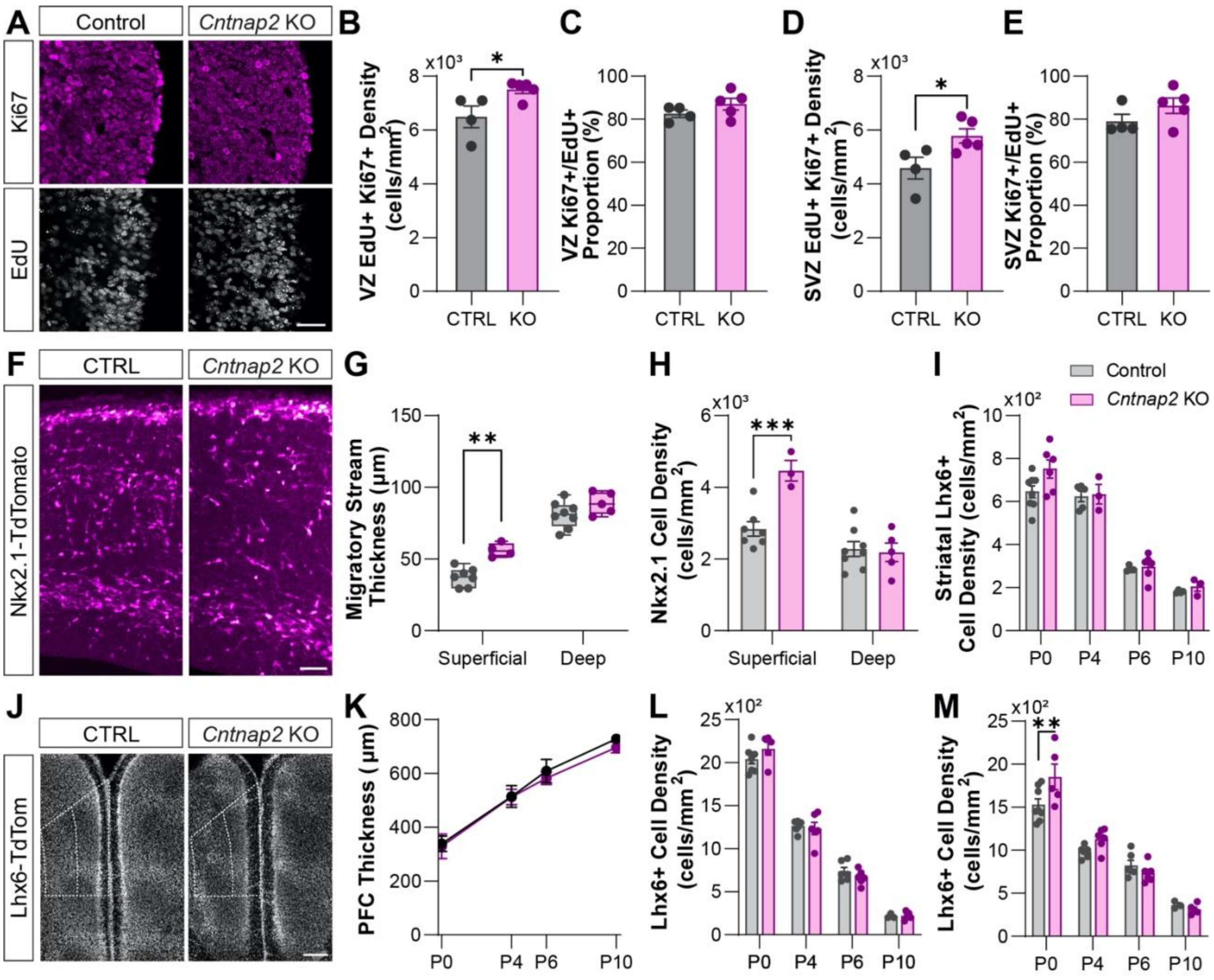

Supplementary Figure 3: Proliferation and migration of the cortical MGE-derived interneurons. **(A)** Ki67 (magenta) and EdU (grey) colocalisation in the VZ and SVZ of the MGE, in Control and the *Cntnap2* knockout, scale: 40 µm. **(B)** EdU+ Ki67+ cell density in the VZ at E14.5 (n = 4 Control, 5 KO, p = 0.035). **(C)** The proportion of EdU+ cells expressing Ki67 in the VZ at E14.5 (n = 4 Control, 5 KO, p = 0.226). **(D)** EdU+ Ki67+ cell density in the SVZ at E14.5 (n = 4 Control, 5 KO, p = 0.038). **(E)** The proportion of EdU+ cells expressing Ki67 in the SVZ at E14.5 (n = 4 Control, 5 KO, p = 0.186). **(F)** MGE derived interneurons (Nkx2.1+, magenta) migrating towards the cortex via two streams, superficial and deep, in Control and *Cntnap2* knockout conditions, scale: 50 µm. **(G)** Thickness of the superficial and deep cortical migratory streams (n = 7 Control, 4 KO, $p_{genotype}$ = 0.0005, $p_{interaction}$ = 0.110, 2-way ANOVA; superficial p = 0.002, deep p = 0.154, Bonferroni post-hoc multiple comparisons). **(H)** Nkx2.1 cell density in the superficial and deep cortical migratory streams in Control and *Cntnap2* KO conditions (n = 7 Control, 4 KO, $p_{genotype}$ = 0.006, $p_{interaction}$ = 0.003, 2-way ANOVA; superficial p = 0.0009, deep p = 0.999, Bonferroni post-hoc multiple comparisons). **(I)** Density of Lhx6+ striatal interneurons across postnatal stages in Control and *Cntnap2* KO conditions (P0: n = 8 Control, 6 KO, p = 0.051; P4: n = 5 Control, 3 KO, p = 0.856; P6: n = 4 Control, 6 KO, p = 0.738; P10: n = 4 Control, 3 KO, p = 0.253).

(J) The prefrontal cortex (PFC, DAPI) in Control and *Cntnap2* KO conditions, separated into upper (nearest to midline) and deeper layers, scale: 200 µm. **(K)** Quantification of the thickness of the PFC in Control and *Cntnap2* KO conditions (P0: n = 8 Control, 4 KO; P4: n = 6 Control, 6 KO; P6: n = 5 Control, 5 KO; P10: n = 4 Control, 4 KO; $p_{genotype} = 0.490$, $p_{interaction} = 0.968$, 2-way ANOVA). **(L-M)** Quantification of cell density in the upper **(L)** and deeper **(M)** layers of the prefrontal cortex in Control and *Cntnap2* KO conditions (P0: n = 8 Control, 5 KO; P4: n = 6 Control, 6 KO; P6: n = 6 Control, 6 KO; P10: n = 3 Control, 5 KO; upper: $p_{genotype} = 0.854$, $p_{interaction} = 0.250$; deeper: $p_{genotype} = 0.106$, $p_{interaction} = 0.015$, 2-way ANOVA; $P0_{deeper}$: p = 0.005, $P4_{deeper}$: p = 0.487, $P6_{deeper}$: p = 0.999, $P10_{deeper}$: p = 0.999, Bonferroni post-hoc multiple comparisons). Unless otherwise specified, a Student's t-test was used to determine significance, p<0.05:*, p<0.01:**, p<0.001:***, p<0.0001:****.

Supplementary Figure 4.

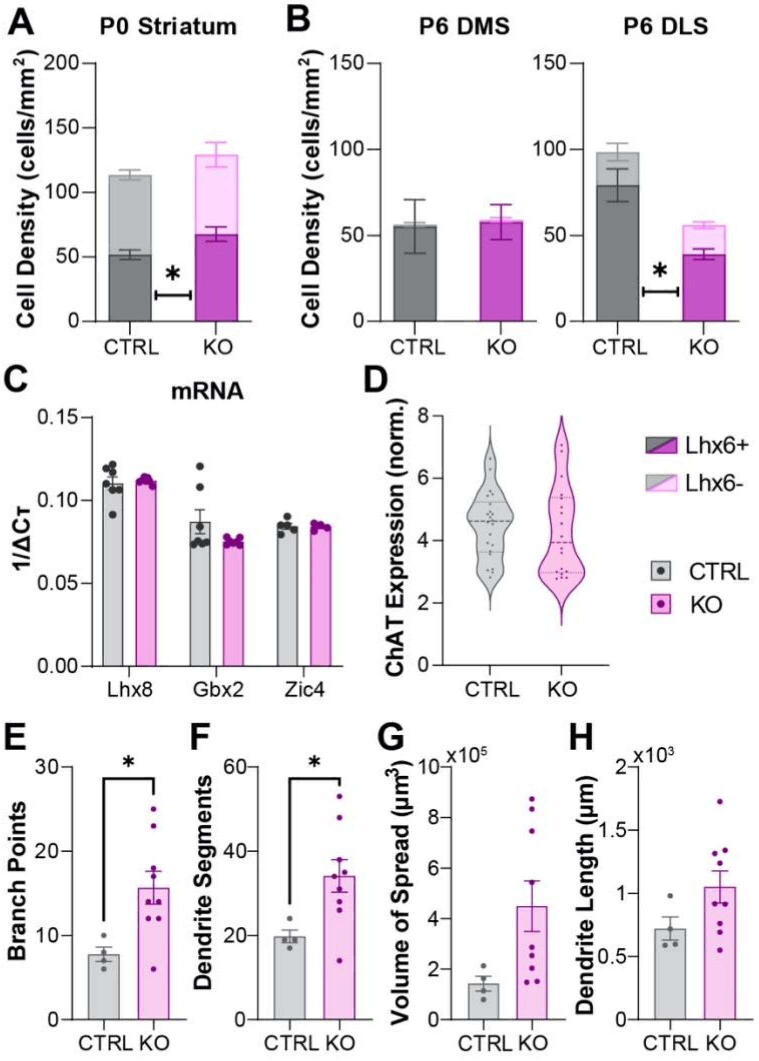

<u>Supplementary Figure 4:</u> **Developmental alterations of the striatal interneurons in the *Cntnap2* KO mice. (A)** Cell density of Lhx6+ ChAT+ and Lhx6- ChAT+ cells, showing the distribution of Lhx6 positive and negative cells within the cholinergic interneuron population, in the striatum at P0 (n = 6 Control, 3 KO, Lhx6+ p = 0.042, Lhx6- p = 0.960). **(B)** Cell density of Lhx6+ ChAT+ and Lhx6- ChAT+ cells in the dorsomedial (DMS; n = 3 Control, 4 KO, p = 0.893 Lhx6+, p = 0.940 Lhx6-) and dorsolateral (DLS; n = 3 Control, 4 KO, p = 0.016 Lhx6+, p = 0.652 Lhx6-) striatum at P6 in Control and *Cntnap2* KO conditions. **(C)** Relative mRNA expression of cholinergic neuron identity related factors: Lhx8 (n = 7 Control, n = 7 KO, p = 0.695), Gbx2 (n = 7 Control, n = 7 KO, p = 0.126), Zic4 (n = 5 Control, n = 4 KO, p = 0.894) in the P6 striatum in Control and *Cntnap2* KO conditions. **(D)** Normalised ChAT protein expression in Lhx6- ChAT+ cells in the striatum at P6 in Control and *Cntnap2* KO conditions (n = 22 cells, 4 Control, 18 cells, 3 KO, p = 0.609). **(E-H)** Dendritic characteristics of striatal cholinergic interneurons at P6 in Control (n = 4) and *Cntnap2* KO (n = 9) conditions: **(E)** Number of branching points (p = 0.025). **(F)** Number of dendritic segments (p = 0.037). **(G)** Volume occupied by dendritic spread (p = 0.075). **(H)** Total dendritic length (p = 0.131). Unless otherwise specified, a Student's t-test was used to determine significance, p<0.05:*, p<0.01:**, p<0.001:***, p<0.0001:****.

Supplementary Figure 5.

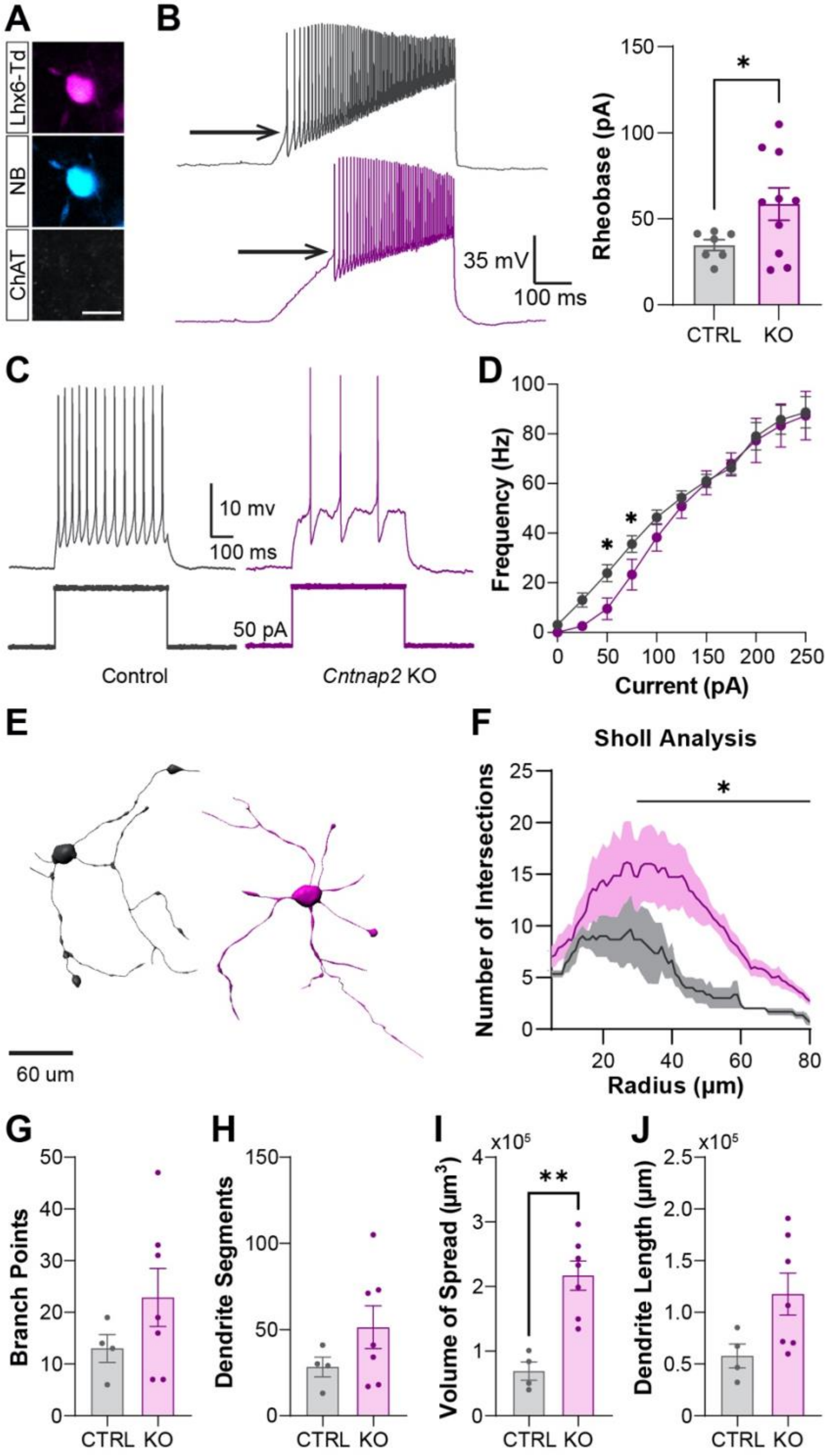

<u>Supplementary Figure 5:</u> Morpho-functional alterations to the Lhx6⁺ GABAergic interneuron population in the *Cntnap2* KO mice. **(A)** ChAT- striatal Lhx6+ interneurons loaded with neurobiotin during electrophysiological recordings at P6 for morphological reconstruction.

(B) Putative fast spiking interneurons (FSIs) responding to a ramp stimulus in Control and *Cntnap2* KO conditions in the striatum at P6, arrow: rheobase. **(C)** Rheobase quantification in putative FSIs in the P6 striatum in Control and *Cntnap2* KO conditions (n = 7 Control, 10 KO, p = 0.035). **(D)** Evoked firing of P6 Lhx6+ putative FSIs. **(E)** Evoked firing rate of putative FSIs in Control and *Cntnap2* KO conditions across increasing current inputs (n = 13 Control, 8 KO, $p_{genotype} = 0.074$, $p_{interaction} = 0.011$, 2-way ANOVA, repeated measures; post-hoc Bonferroni comparisons: 50 pA p = 0.014, 75 pA p = 0.035). **(F)** Reconstructed ChAT- Lhx6+ striatal interneurons. **(G)** Sholl analysis measuring the number of dendrites intersecting circles increasing in radius by 1 µm (n = 3 Control, 7 KO, $p_{genotype} = 0.112$, $p_{interaction} = 0.176$, 2-way ANOVA). **(H-K)** Dendritic characteristics of striatal Lhx6+ ChAT- interneurons at P6 in Control (n = 4) and *Cntnap2* KO (n = 7) conditions: **(H)** Volume occupied by dendritic spread (p = 0.001). **(I)** Number of branching points (p = 0.240). **(J)** Number of dendritic segments (p = 0.215). **(K)** Total dendritic length (p = 0.068). Unless otherwise specified, a Student's t-test was used to determine significance, p<0.05:*, p<0.01:**, p<0.001:***, p<0.0001:****.

Supplementary Figure 6.

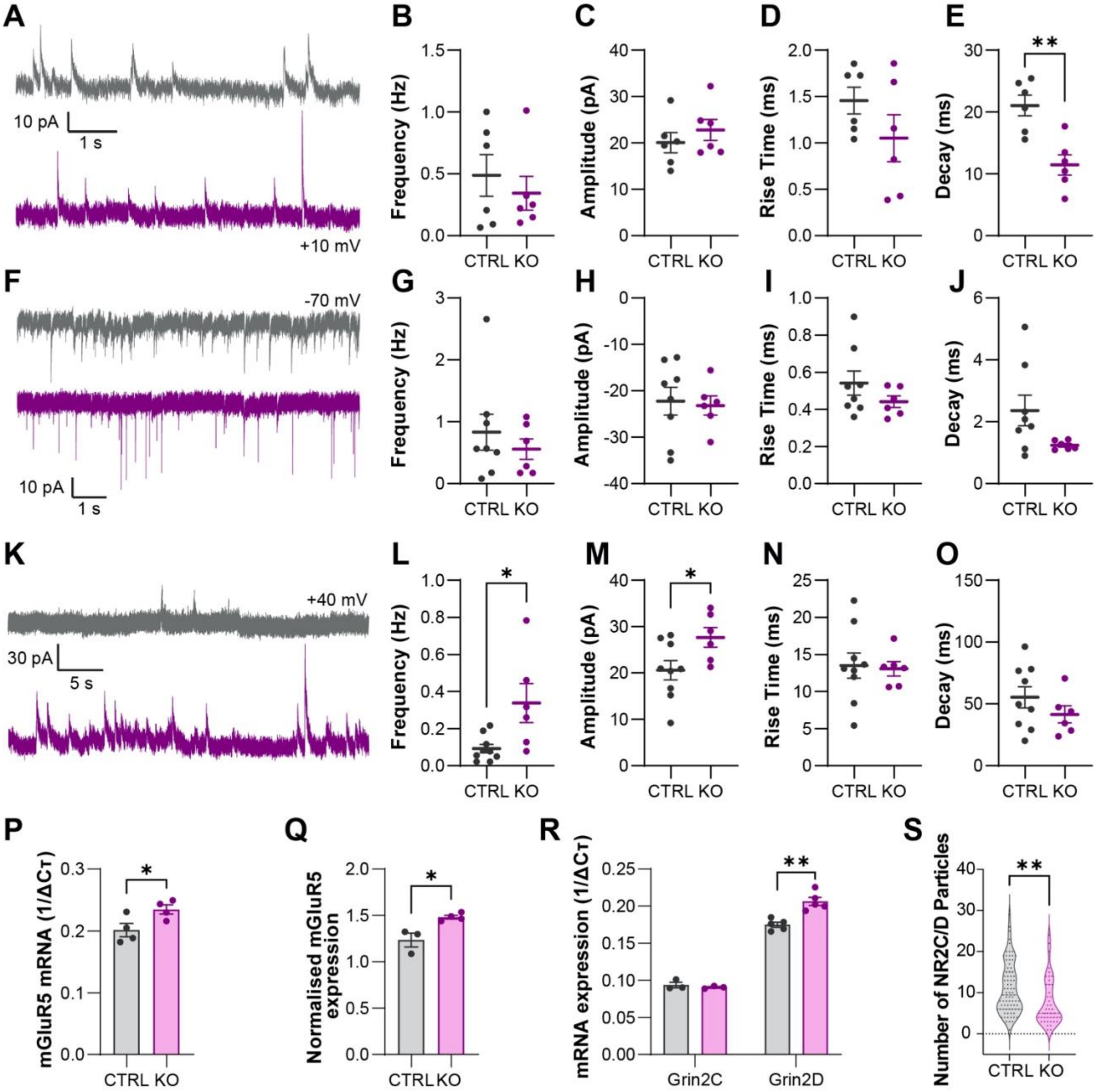

<u>Supplementary Figure 6:</u> Glutamatergic and GABAergic synaptic inputs onto Lhx6⁺ GABAergic interneuron in the *Cntnap2* KO mice. **(A)** Example trace of spontaneous GABA$_A$-driven inhibitory post-synaptic currents (sIPSC) recorded in voltage-clamp at 0 mV in Lhx6+ interneurons in control and *Cntnap2* KO conditions (n = 6 Control, 6 KO cells). **(B)** sIPSC Frequency (p = 0.522). **(C)** sIPSC Amplitude (p = 0.406). **(D)** sIPSC Rise time (p = 0.194). **(E)** sIPSC Decay time (p = 0.002). **(F)** Example trace of spontaneous AMPA/KA-driven excitatory post-synaptic currents (sEPSC) recorded in voltage-clamp at -60 mV in Lhx6+ interneurons in control and *Cntnap2* KO conditions (n = 8 Control, 6 KO cells). **(G)** sEPSC Frequency (p = 0.976). **(H)** sEPSC Amplitude (p = 0.478). **(I)** sEPSC Rise time (p = 0.153). **(J)** sEPSC Decay time (p = 0.097).

(K) Example trace of spontaneous NMDA-driven excitatory post-synaptic currents (sEPSC) recorded in voltage-clamp at +40 mV in Lhx6+ interneurons in control and *Cntnap2* KO conditions (n = 9 Control, 6 KO cells). **(L)** sEPSC Frequency (p = 0.016). **(M)** sEPSC Amplitude (p = 0.038). **(N)** sEPSC Rise time (p = 0.847). **(O)** sIPSC Decay time (p = 0.268). **(P)** *Grm5* mRNA levels in FACS-sorted Lhx6+ interneurons in control and *Cntnap2* KO conditions (n = 4 control, 4 KO; p = 0.045). **(Q)** mGlur5 protein levels from immunostaining (n = 3 control, 4 KO; p = 0.014). **(R)** *Grin2C* (n = 3 control, 3 KO; p = 0.0.484) and *Grin2D* mRNA levels (n = 5 control, 5 KO; p = 0.001) in FACS-sorted Lhx6+ interneurons in control and *Cntnap2* KO conditions. **(S)** NMDAR2C/D protein expression from immunostaining analysis (n = 78 control cells, 43 KO cells; p = 0.004). Unless otherwise specified, a Student's t-test was used to determine significance, p<0.05:*, p<0.01:**, p<0.001:***, p<0.0001:****.

2 Tables

Lhx6+ Cholinergic Interneurons					
Property	Control		KO		*p*
	Mean	n	Mean	n	
Resting membrane potential (mV)	-44.90 ± 1.55	16	-47.44 ± 2.27	12	0.368
Input resistance (MΩ)	551.24 ± 60.17	14	408.61 ± 23.79	12	0.049
Membrane capacitance (pF)	596.63 ±59.82	14	802.39 ± 125.74	12	0.135
Sag ratio	0.27 ± 0.03	10	0.30 ± 0.07	7	0.676
Spike adaptation index	0.60 ± 0.05	14	0.60 ± 0.05	11	0.926
Latency to first spike (ms)	19.64 ± 3.13	14	21.59 ± 3.22	11	0.671
Action potential threshold (mV)	-35.59 ± 1.18	15	-36.58 ± 1.97	12	0.655
Action potential amplitude (mV)	51.95 ± 2.13	15	50.13 ± 3.62	12	0.654
Action potential rise time (ms)	1.07 ± 0.09	15	0.77 ± 0.06	12	0.017
Action potential decay (ms)	2.05 ± 0.01	15	2.02 ± 0.02	12	0.229
Action potential halfwidth (ms)	2.71 ± 0.16	15	2.50 ± 0.14	12	0.347
Action potential fast AHP (mV)	9.02 ± 1.23	15	6.11 ± 1.35	12	0.126
Action potential medium AHP (mV)	-13.77 ± 1.22	15	-13.78 ± 1.00	12	0.993

Table 1: Intrinsic properties of Lhx6+ striatal cholinergic interneurons in the control and *Cntnap2* KO mice at P6.

AHP: Afterhyperpolarization. Statistics from two-sample Student’s t-test.

Lhx6⁺ Putative Fast Spiking Interneurons					
	Control		KO		p
Property	Mean	n	Mean	n	
Resting membrane potential (mV)	-54.45 ± 1.50	22	-53.98 ± 3.09	13	0.880
Input resistance (MΩ)	780.28 ± 75.02	19	406.55 ± 47.03	14	0.0005
Membrane capacitance (pF)	805.33 ± 126.76	19	1064.36 ± 273.87	14	0.357
Latency to first spike (ms)	270.01 ± 35.37	6	315.87 ± 25.58	10	0.303
Action potential threshold (mV)	-35.49 ± 1.43	19	-32.45 ± 1.91	11	0.184
Action potential amplitude (mV)	48.15 ± 1.80	19	44.59 ± 3.43	11	0.320
Action potential rise time (ms)	0.72 ± 0.05	19	0.59 ± 0.05	11	0.078
Action potential decay (ms)	1.80 ± 0.05	19	1.59 ± 0.09	11	0.031
Action potential halfwidth (ms)	1.74 ± 0.12	19	1.22 ± 0.11	11	0.006
Action potential fast AHP (mV)	-2.69 ± 1.72	19	-10.89 ± 1.09	11	0.002
Action potential medium AHP (mV)	-13.15 ± 0.92	19	-15.37 ± 0.70	11	0.105

Table 2: Intrinsic properties of Lhx6+ striatal GABAergic fast-spiking interneurons in the control and *Cntnap2* KO mice at P6.

AHP: Afterhyperpolarization. Statistics from two-sample Student’s t-test.
